# A Limit-Aware Sparse Frequency-Domain Decision Engine for EMI Risk Feedback in Resource-Constrained Systems

**DOI:** 10.3390/s26134197

**Published:** 2026-07-02

**Authors:** Jiaxuan Hu, Weiqi Luo, Kaiwen Xiao, Yingping Chen

**Affiliations:** 1 College of Integrated Circuits & Micro-Nano Electronics, Fudan University, Shanghai 200433, China; 23307130329@m.fudan.edu.cn (J.H.); 23307130308@m.fudan.edu.cn (W.L.); 23301020008@m.fudan.edu.cn (K.X.); 2 Frontier Institute of Chip and System, Fudan University, Shanghai 200433, China

**Keywords:** electromagnetic interference (EMI), GaN power converter, EMI risk feedback, sparse Fourier transform (SFT), selective frequency-domain decision, limit-aware risk decision, RTL implementation

## Abstract

Resource-constrained electromagnetic interference (EMI) management requires a frequency-domain feedback path, while FFT-based full-spectrum processing introduces redundant computation, storage, and data movement for decision tasks. This paper proposes a limit-aware sparse frequency-domain decision engine for internal EMI risk feedback. The engine redefines EMI analysis from spectrum reconstruction to selective exceedance verification and uses randomized spectral reordering, flat-window bucket aggregation, and folded sampling to compress the length-*N* spectral search into bucket-level observations. Then, by comparing bucket-level amplitude envelopes with local limit envelopes, the method excludes risk-negative buckets, and only uncertain buckets are further refined through phase localization and sequential verification. Degradation experiments involving continuous background uplift, main-harmonic sidebands, and parasitic resonance clusters clarify the applicability boundary of the proposed method, and measured GaN power-converter spectra acquired through an in situ EMI sensing chain remain inside the empirical usable region. RTL evaluation at 100 MHz shows that the proposed design achieves an average decision latency of 6.031 ms. Compared with two FFT baseline implementations, it reduces BRAM usage by 95.17% and 97.59%, dynamic power by 54.0% and 83.0%, and per-decision dynamic energy by 46.3× and 33.3×, respectively. The results show that the proposed decision engine reduces hardware overhead for frequency-domain EMI risk feedback in resource-constrained systems.

## 1. Introduction

### 1.1. Research Background and Problem Statement

In automotive power supplies, consumer electronics, and high-frequency power conversion, electromagnetic compatibility has become an important design constraint [1,2]. It affects power-chip design, package and PCB implementation, and system integration. In product-level EMC testing, conducted-emission spectra are usually compared with standard limit curves. When the emission level is close to or above the limit range, the product often requires redesign, repeated testing, and parameter tuning. This increases development cost and lengthens the time to market.

Traditional EMI debugging usually uses a spectrum analyzer or an EMI receiver to obtain frequency-domain information. Engineers then adjust design parameters according to the observed spectrum and the corresponding limit curve. This procedure is suitable for standard testing and engineering diagnosis, but it is slow and dependent on external equipment. It is also difficult to use it as an online feedback path for continuously tracking EMI variations during chip operation.

Recent studies have explored the integration of EMI sensing and regulation into chips or power systems. Spectral information can then be used to update modulation parameters online. This can reduce the dependence on external test equipment and manual tuning during regulation. An early example is the autocovariance-based on-chip power-supply noise spectrum measurement method proposed by Alon, Stojanović, and Horowitz in 2005 [3]. In 2025, Chen, Yuan, and Liu proposed a closed-loop EMI-regulated gallium nitride (GaN) power converter with in situ EMI sensing and global excess-spectrum modulation (GESM) [4]. These studies show that online EMI management is feasible at the system level.

However, the online spectral-analysis backend in these systems still relies on conventional FFT-based processing. DFT/FFT remains a common frequency-domain tool in modern digital sensor and RF-receiver designs, and it is also widely used on FPGA platforms. Repeated long-sequence FFT computations can introduce considerable computational latency, storage access, and data movement. Multi-resolution FFT computations can also bring high time cost [5]. When the frequency-domain analysis stage is moved toward an on-chip or other resource-constrained implementation, the power, area, memory, and timing pressure of FFT-based full-spectrum processing become major bottlenecks.

**Figure 1:**
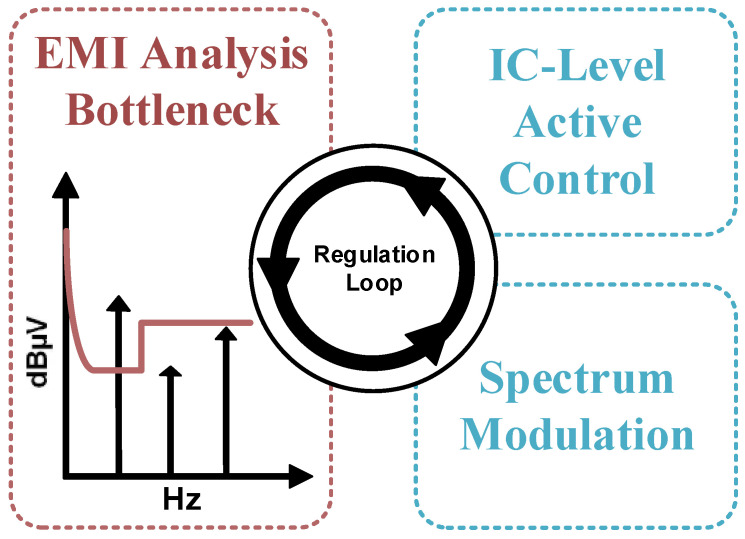
Conceptual EMI risk-feedback architecture with a frequency-domain decision backend.

Therefore, this paper focuses on one practical question: can a lower-cost frequency-domain decision backend be built to quickly extract risk-related frequency-domain variables for internal EMI risk feedback, including the internal risk state, worst-margin estimate, and candidate risk locations, and provide feedback for the EMI management system shown in Figure 1?

### 1.2. International Research Status and Motivation

For the computational complexity and resource cost in on-chip spectral analysis discussed in Section 1.1, existing studies mainly follow two directions. The first direction improves algorithms inside the traditional Fourier-analysis framework. Duhamel and Hollmann generalized the split-radix FFT in 1984 [6]. Sreenivas and Rao proposed FFT pruning for both input and output in 1979 [7]. Frigo and Johnson described the design and implementation of FFTW3 in 2005 [8]. These methods can reduce some cost. Their main task is still to compute a full or nearly full spectrum. Their asymptotic complexity still does not fundamentally break the O(NlogN) bound [6,7,8,9,10,11].

The second direction goes outside the standard FFT flow. It uses transient features or sparse time–frequency structures of EMI signals for decomposition and identification. Huang et al. proposed empirical mode decomposition (EMD) and the Hilbert–Huang spectrum in 1998 [12]. Azpúrua, Pous, and Silva proposed a time-domain EMI decomposition method in 2016 [13]. Wavelet transform [14,15], sparse representation [16,17], and adaptive sparse time–frequency analysis [18,19,20] have also been used for EMI source identification and fault diagnosis. These methods are useful for understanding signal components and interference mechanisms [13,14,16,18,21,22]. But they are less direct for generating frequency-domain sensing variables for control feedback.

Compared with these time–frequency decomposition methods, the sparse fast Fourier transform (SFT/sFFT) is more direct for frequency-domain analysis. Its output directly gives frequency-domain amplitudes. This makes it a possible choice under limited hardware resources. In 2012, Hassanieh, Indyk, Katabi, and Price proposed *A Simple and Practical Algorithm for Sparse Fourier Transform* [23]. In the same year, they proposed *Nearly Optimal Sparse Fourier Transform* [24]. These two works gave better complexity results than the conventional FFT for exactly *k*-sparse and general sparse cases. But studies that directly use sFFT for on-chip closed-loop EMI decisions are still limited [25,26].

Li, Hou, Jiang, and Chen classified the existing sFFT methods in 2022. They divided these methods by the bucketization filter. The three types are Dirichlet-kernel methods, flat-window methods, and aliasing-filter methods [27].

The first typical type is the randomized sparse Fourier method based on a Dirichlet-kernel filter bank. A representative example is the Ann Arbor fast Fourier transform (AAFFT) series. It was mainly developed by Gilbert, Iwen, Strauss, and others [28,29]. This type is an early basic framework in sparse Fourier analysis. Its parameters are hard to tune. Its multi-round iterations also make implementation more difficult and increase run-time overhead.

The second type is the MIT sFFT series based on a flat-window filter. Around 2012, Hassanieh, Indyk, Katabi, and Price proposed sFFT1.0 and sFFT2.0. These methods use multi-round random hashing and a voting mechanism to recover candidate frequencies. They have good bucket-collision tolerance. They also need several hashing rounds, candidate-table updates, and voting aggregation. This brings high storage-access and control overhead [23]. sFFT3.0 and sFFT4.0 use phase estimation or phase decoding. They recover the in-bucket frequency location from phase differences between observations under different time shifts [24].

In summary, the existing EMI detection methods still mainly use FFT-type spectral estimation. Non-FFT signal decomposition methods can describe interference structure. They are not directly linked to mapped-reference-limit decisions. sFFT has clear computational advantages for sparse spectral analysis. But its use in EMC is still limited. It still has room for further development. Existing sFFT methods have different technical details. Most of them still try to obtain the spectrum efficiently. They recover the spectrum first and make a decision afterward. And computation is still used on spectral components that do not directly affect the final decision. For on-chip closed-loop EMI regulation, the system does not need the full spectrum. It needs the risk state of the current spectrum relative to the limit curve. It also needs the worst exceedance level and the trend of that level. The frequency-domain analysis flow should be rebuilt from a control-oriented view, not a reconstruction-oriented view.

## 2. Limit-Aware Decision Modeling for Internal EMI Risk Feedback

For data processing, this paper takes the output of a calibrated EMI sensing chain as the input to the frequency-domain decision algorithm. This input is not restricted to a specific external test fixture. Instead, it refers to a digital observation that has passed through front-end sensing, quantization, and calibration and can be mapped to a discrete frequency-domain limit reference. Let the EMI-related voltage to be observed be $v_{\mathrm{EMI}}\left(t\right)$, the equivalent frequency-domain transfer function of the sensing chain be $H_{s}\left(f\right)$, the corresponding time-domain response be $h_{s}\left(t\right)$, and the residual error be $n_{s}\left(t\right)$. The analog signal after front-end sensing can be expressed as

(1) $$v_{s}\left(t\right)=h_{s}\left(t\right)*v_{\mathrm{EMI}}\left(t\right)+n_{s}\left(t\right).$$

After sampling and quantization, the raw digital code stream is obtained as

(2) $$q\left[n\right]=Q\{v_{s}\left(nT_{s}\right)\},$$

where Q{·} denotes the sample-and-hold, quantization, and digital encoding processes. The measured EMI data used in this paper are calibrated and mapped into the frequency domain with reference to the in situ EMI sensing chain reported by Chen et al. [4]. In that chain, the EMI-related voltage $V_{\mathrm{EMI}}$ is sensed through a coupling capacitor $C_{\mathrm{EMI}}$. Two sampling channels driven by randomized sampling clocks then generate $Q_{\mathrm{EMI}0}$ and $Q_{\mathrm{EMI}1}$, which represent $V_{\mathrm{EMI}}\left(t\right)$ and $V_{\mathrm{EMI}}\left(t+\tau \right)$, respectively. Chen et al. further use these two code streams to calculate the autocovariance sequence $R_{\mathrm{EMI}}\left(\tau \right)$, from which an online EMI spectral observation is obtained through frequency-domain transformation.

The decision backend in this work does not directly take $R_{\mathrm{EMI}}\left(\tau \right)$ as the algorithm input. Instead, it uses the calibrated time-domain code stream as the object of sparse frequency-domain search. Specifically, the raw code stream q[n] is first processed by quantization-scale conversion, DC-offset correction, front-end gain compensation, and sensing-chain frequency-response calibration, yielding an amplitude-equivalent time-domain sequence

(3) $$u\left[n\right]=C_{s}\left\{q\left[n\right]\right\},$$

where $C_{s}\left\{\cdot \right\}$ denotes the digital calibration operator obtained from front-end characterization, including code-to-voltage conversion and compensation for $H_{s}\left(f\right)$. A steady-state operating window is then selected. The DC component and slowly varying trend are removed, and a lightweight window function w[n] is applied. The discrete-time input used by the following algorithm is therefore

(4) $$x\left[n\right]=w\left[n\right]\left(u\left[n+n_{0}\right]-\hat{b}\left[n\right]\right),\;n=0,1,\ldots ,N-1,$$

where $n_{0}$ is the starting index of the steady-state window, and $\hat{b}\left[n\right]$ denotes the estimated DC component and low-frequency drift. Previous measurement results show that the on-chip sensing chain reported by Chen et al. achieves a 500 MHz sensing bandwidth, 1 mV sensing accuracy, and 9 kHz frequency resolution [4]. A standard EMI measurement is not defined only by a spectral-amplitude sequence and a limit curve. The CISPR 16 receiver framework specifies the characteristics and performance of radio-disturbance measuring apparatus, including receiver-related requirements such as selectivity, detector behavior, and response to pulses [30]. Therefore, it should still be noted that this input is used only to generate internal risk decisions and regulation feedback. It is not the final test spectrum obtained by a standard EMI receiver under the prescribed detector, IF bandwidth, dwell time, and sweep procedure [31].

### 2.1. Reference Limit Mapping for Internal EMI Risk Decision

To construct an internal EMI risk variable for closed-loop regulation, this paper first maps a conducted-emission reference limit curve to the discrete frequency domain of the decision engine. The EN 55032 Class B conducted-emission quasi-peak limit is used as the numerical reference curve in this work [31]. This standard limit is adopted not to perform a final regulatory compliance test but to provide a public, reproducible, and frequency-dependent risk reference for the following algorithm. Table 1 lists the reference limit values used for the discrete mapping.

This paper uses the quasi-peak reference curve as a numerical risk reference. It reflects the practical effect of repetitive switching interference. According to the mapped reference limit, the limit in the 0.15 MHz to 30 MHz band can be written as a piecewise function $L_{\mathrm{dB}\mu V}\left(f\right)$.

To map this numerical reference curve to discrete spectral amplitudes, the limit is first converted to the linear voltage domain. The mapped reference limit can then be written as a discrete spectral-domain reference function. It has the same dimension as the discrete frequency-domain signal |X[k]|:

(5) $$L\left[k\right]=\left\{\begin{array}{cc} \frac{N}{\sqrt{2}}10^{L_{\mathrm{dB}\mu V}\left(f_{k}\right)/20}\times 10^{-6}, & f_{k}\in \left[0.15,\;30\right]\;\mathrm{MHz},\\ +\infty , & \mathrm{otherwise}. \end{array}\right.$$

### 2.2. Task Redefinition and Construction of a Frequency-Domain Exceedance Indicator

After the limit curve is discretized, the discrete frequency-index set for the band of interest is defined as $\Psi =\{k\in \left\{0,1,\ldots ,N-1\right\}|f_{k}\in \left[0.15,\;30\right]\;\mathrm{MHz}\}$. In the frequency-domain exceedance-indication framework used in this paper, the risk constraint in this band can be written as:

(6) |X[k]|≤L[k],∀k∈Ψ.

To describe how much each frequency bin deviates from its limit, the pointwise exceedance margin is defined as $\Delta_{X}\left(k\right)≜\left|X\left[k\right]\right|-L\left[k\right]$. The global worst-case margin is defined as $m\left(X\right)≜\underset{k\in \Psi }{\sup}\Delta_{X}\left(k\right)$.

The worst exceedance-indication result is then written as:

(7) $$\left\{\begin{array}{cc} m(X)\leq 0, & \mathrm{risk}-\mathrm{negative},\\ m(X)>0, & \mathrm{risk}-\mathrm{positive}. \end{array}\right.$$

Here, risk-negative means that the observed spectrum does not show a positive pointwise margin under the discrete limit mapping used in this paper. Risk-positive means that there is at least one positive pointwise risk margin in the band of interest. This risk state can drive the later spread-spectrum modulation. m(X) is the risk state variable in closed-loop regulation. It shows the worst deviation of the current spectrum from the mapped reference limit. It also gives feedback for the next modulation strategy. The original pointwise constraint system is changed into a decision problem controlled by the worst exceedance amount. Many frequency bins with low margins do not affect m(X). They do not need to be checked in detail. The number of frequency bins that need accurate amplitude computation is greatly reduced.

It should also be noted that this paper does not try to reproduce the full measurement procedure of an EMI receiver. The target is an internal EMI risk-feedback path for closed-loop EMI regulation. In this setting, the main spectral peak is not exactly equal to the receiver reading. The two are still strongly correlated. Chen, Yuan, and Liu also show that online spectral analysis can differ from a commercial spectrum analyzer by up to about 3 dB [4]. This shows a measurement–definition difference between standard receiver readings and discrete-spectrum approximations. The output of this paper should be understood as an internal risk-decision metric for engineering regulation and fast screening. It is not a final decision for standard certification. Samples close to the limit boundary still need to be checked by the standard EMI receiver procedure.

## 3. Proposed Limit-Aware Sparse Frequency-Domain Analysis Method for Feedback Generation

### 3.1. Randomized Spectral Reordering and Flat-Window Bucket Observations

For the conducted EMI spectrum studied in this paper, a best *K*-term approximation is used. Here, sparsity means that the spectrum can be well described by a small number of dominant components. The inband optimal *K*-term approximation support is defined as:

(8) $$\Lambda_{K}\in \underset{\Lambda \subseteq \Psi ,\;|\Lambda |=K}{\arg\;\inf}{∥{X|}_{\Psi }{-X|}_{\Lambda }∥}_{2}.$$

The corresponding dominant spectrum and tail spectrum are written as:

(9) $$X_{K}\left[k\right]≜\left\{\begin{array}{cc} X[k], & k\in \Lambda_{K},\\ 0, & k\notin \Lambda_{K}, \end{array}\right.\;$$

(10) $$R_{K}\left[k\right]≜X\left[k\right]-X_{K}\left[k\right],\;k\in \Psi .$$

Random permutation parameters γ and σ are then chosen, where:

(11) γ∈{1,2,…,N−1},gcd(γ,N)=1,σ∈{0,1,…,N−1}.

Let η be the inverse of γ modulo *N*. The frequency-domain randomized reordering operator $P_{\gamma ,\sigma }$ acting on X[k] is defined as:

(12) $$X_{\gamma ,\sigma }\left[k\right]≜\left(P_{\gamma ,\sigma }X\right)\left[k\right]=X\left[\left(\eta k+\sigma \right)\;\mathrm{mod}\;N\right],\;k=0,1,\ldots ,N-1.$$

Accordingly, $X_{K,\gamma ,\sigma }≜P_{\gamma ,\sigma }X_{K}$ and $R_{K,\gamma ,\sigma }≜P_{\gamma ,\sigma }R_{K}$ are obtained. Some dominant components may be close to each other in the original frequency domain. This reordering moves them to different positions. It reduces the chance that they gather in the same local observation window. The corresponding time-domain implementation can be written as:

(13) $$x_{\gamma ,\sigma }\left[n\right]=x\left[\left(\gamma n\right)\;\mathrm{mod}\;N\right]W_{N}^{\gamma \sigma n},\;n=0,1,\ldots ,N-1.$$

After spectral reordering, a flat window g[n] with length *N* is introduced. Its discrete Fourier transform is denoted by G[k]. G[k] should have an approximately rectangular passband. It should also have controlled stopband leakage. The detailed construction can follow the work of Hassanieh, Indyk, Katabi, and Price at SODA 2012 [23] and is not repeated here.

Let

(14) $$z\left[n\right]≜g\left[n\right]x_{\gamma ,\sigma }\left[n\right],\;\;\;\;\;n=0,1,\ldots ,N-1,$$

Let its *N*-point discrete Fourier transform be Z[k]. Multiplication in the time domain corresponds to circular convolution in the frequency domain. Thus, Z[k] is the convolution of $X_{\gamma ,\sigma }\left[k\right]$ and G[k].

Let the number of buckets be B≪N. Define STEP≜N/B. To further reduce the observation dimension, z[n] is periodically folded in time to length *B*. The folded sequence is denoted by $\tilde{z}\left[r\right]$. Let Y[b] be the *B*-point discrete Fourier transform of $\tilde{z}\left[r\right]$. Time-domain folding corresponds to equally spaced sampling in the frequency domain. We then have:

(15) Y[b]=Z[b·STEP],b=0,1,…,B−1.

Define the candidate frequency set corresponding to bucket b as:

(16) $$S_{b}≜\left\{k=\left(b\cdot \mathrm{STEP}+a\right)\;\mathrm{mod}\;N|a\in \left[-\frac{\mathrm{STEP}}{2},\frac{\mathrm{STEP}}{2}\right)\cap Z\right\}.$$

If there is an offset $a_{b}\in \left[-\mathrm{STEP}/2,\mathrm{STEP}/2\right)\cap Z$ in bucket *b*, and $|X_{\gamma ,\sigma }\left[b\cdot \mathrm{STEP}+a_{b}\right]|$ is much larger than the other terms in $S_{b}$, the bucket satisfies the approximate single-tone dominance condition. In this case, the bucket observation can be written as:

(17) $$Y\left[b\right]=X_{\gamma ,\sigma }\left[b\cdot \mathrm{STEP}+a_{b}\right]G\left[-a_{b}\right]+\varepsilon_{b},$$

The perturbation term $\varepsilon_{b}$ means the error from weak in-bucket components and out-of-bucket leakage. Computing $a_{b}$ needs relatively high cost. It would reduce the time and resource efficiency of later limit-aware decisions. The next section shows how to build reliable upper and lower envelopes for the dominant spectral-line amplitude when $a_{b}$ is unknown. It only uses the boundary information of Y[b] and G[−a].

### 3.2. Construction of Bucket-Level Amplitude Envelopes and Local Limit Envelopes

As noted in Section 3.1, $\varepsilon_{b}$ contains the total perturbation from in-bucket non-dominant components and out-of-bucket leakage. To keep the argument general, a nonnegative upper bound $\nu_{b}\geq \left|\varepsilon_{b}\right|$ is introduced. It is treated as the perturbation margin of bucket *b*. The triangle inequality gives:

(18) $${\left(|Y\left[b\right]-\nu_{b}|\right)}_{+}\leq \;|X_{\gamma ,\sigma }\left[b\cdot \mathrm{STEP}+a_{b}\right]|\;|G\left[-a_{b}\right]|\leq |Y\left[b\right]|+\nu_{b}.$$

Here, ${\left(u\right)}_{+}≜\max\left\{u,0\right\}$. For simplicity, the following derivation assumes $\nu_{b}=0$. Section 4.2 will show that this is a reasonable engineering approximation. Since $a_{b}$ is unknown, the exact value of $G[-a_{b}]$ is also unknown. Its range is controlled by the upper and lower response bounds of the flat window over the local candidate set. For notational compactness, define A=[−STEP/2,STEP/2)∩Z. The amplitude of the dominant spectral line satisfies the following two-sided envelope:

(19) $$\frac{\left|Y[b]\right|}{\underset{a\in A}{\sup}\left|G\left[-a\right]\right|}\leq \left|X_{\gamma ,\sigma }\left[b\cdot \mathrm{STEP}+a_{b}\right]\right|\leq \frac{\left|Y[b]\right|}{\underset{a\in A}{\inf}\left|G\left[-a\right]\right|}.$$

Next, consider the limit side. The original limit function L[k] is mapped to the reordered domain by the same rule. The reordered limit sequence is defined as $L_{\gamma ,\sigma }\left[k\right]$. The original band of interest Ψ is mapped to $\Psi_{\gamma ,\sigma }$ in the reordered domain. For bucket *b*, the EMI decision does not use the whole $S_{b}$. It only uses the part shared by $S_{b}$ and the reordered band of interest. Define $S_{b}^{\Psi }≜S_{b}\cap \Psi_{\gamma ,\sigma }$. If $S_{b}^{\Psi }=⌀$, the bucket has no candidate frequency related to the band of interest in the reordered domain. The following discussion only considers buckets with $S_{b}^{\Psi }\neq ⌀$. For these valid buckets, the true dominant offset $a_{b}$ is still unknown. The local limit $L_{\gamma ,\sigma }\left[b\cdot \mathrm{STEP}+a_{b}\right]$ cannot be obtained directly. Only upper and lower envelopes can be built on $S_{b}^{\Psi }$. If the true dominant offset of bucket *b* corresponds to $k_{b}^{*}=b\cdot \mathrm{STEP}+a_{b}\in \Lambda_{K}^{\gamma ,\sigma }\cap S_{b}^{\Psi }$, then:

(20) $$\underset{k\in S_{b}^{\Psi }}{\inf}L_{\gamma ,\sigma }\left[k\right]\leq L_{\gamma ,\sigma }\left[k_{b}^{*}\right]\leq \underset{k\in S_{b}^{\Psi }}{\sup}L_{\gamma ,\sigma }\left[k\right].$$

After the bucket-level amplitude envelope and the local limit envelope are built, the comparison is changed. It is no longer a direct comparison between an unknown dominant spectral line and its unknown local limit. It becomes a comparison between two intervals.

A traditional FFT flow computes |X[k]| point by point. It then compares |X[k]| with L[k]. Here, point estimation is changed into set-valued estimation. A confidence interval containing the true spectral value is built. This avoids direct recovery of the frequency location and amplitude. If the amplitude envelope and the limit envelope are already separated as intervals, the risk decision can be made directly. The exact frequency bin and amplitude do not need to be recovered. In-bucket refinement is needed only when the two intervals still overlap.

### 3.3. Bucket-Level Risk Certification for Limit-Aware Decisions

After random reordering, the image of the optimal *K*-term support $\Lambda_{K}$ in the reordered domain is denoted as $\Lambda_{K}^{\gamma ,\sigma }$. The set of single-tone valid buckets is defined as:

(21) $$\Omega_{\mathrm{iso}}≜\left\{b\in \left\{0,1,\ldots ,B-1\right\}||\Lambda_{K}^{\gamma ,\sigma }\cap S_{b}^{\Psi }|=1\right\}.$$

For any valid bucket $b\in \Omega_{\mathrm{iso}}$ with $S_{b}^{\Psi }\neq ⌀$, the true dominant spectral-line amplitude cannot be obtained point by point. Its corresponding local limit also cannot be obtained point by point. Both values still lie in two computable intervals. The amplitude-envelope interval and the local-limit-envelope interval of bucket *b* are defined as:

(22) $$A_{b}≜\left[\frac{\left|Y[b]\right|}{\underset{a\in A}{\sup}\left|G\left[-a\right]\right|},\frac{\left|Y[b]\right|}{\underset{a\in A}{\inf}\left|G\left[-a\right]\right|}\right],$$

(23) $$L_{b}≜\left[\underset{k\in S_{b}^{\Psi }}{\inf}L_{\gamma ,\sigma }\left[k\right],\underset{k\in S_{b}^{\Psi }}{\sup}L_{\gamma ,\sigma }\left[k\right]\right].$$

The core of bucket-level certification is to check the relative position of the two intervals $A_{b}$ and $L_{b}$. Based on this, the bucket-level exceedance-certification statement is first given. If, for a valid bucket *b*,

(24) $$\frac{\left|Y[b]\right|}{\underset{a\in A}{\sup}\left|G\left[-a\right]\right|}>\underset{k\in S_{b}^{\Psi }}{\sup}L_{\gamma ,\sigma }\left[k\right],$$

then the envelope relation gives:

(25) $$|X_{\gamma ,\sigma }\left[k_{b}^{*}\right]|\geq \frac{\left|Y[b]\right|}{\underset{a\in A}{\sup}\left|G\left[-a\right]\right|}>\underset{k\in S_{b}^{\Psi }}{\sup}L_{\gamma ,\sigma }\left[k\right]\geq L_{\gamma ,\sigma }\left[k_{b}^{*}\right].$$

By the definition of the reordering map, let ${\tilde{k}}_{b}≜\left(\eta k_{b}^{*}+\sigma \right)\;\mathrm{mod}\;N$.

Then ${\tilde{k}}_{b}\in \Psi$, and $|X\left[{\tilde{k}}_{b}\right]\left|=\right|X_{\gamma ,\sigma }\left[k_{b}^{*}\right]>L_{\gamma ,\sigma }\left[k_{b}^{*}\right]=L\left[{\tilde{k}}_{b}\right]$. Therefore,

(26) $$m\left(X\right)=\underset{k\in \Psi }{\sup}\left(|X\left[k\right]\right|-L\left[k\right])>|X\left[{\tilde{k}}_{b}\right]|-L\left[{\tilde{k}}_{b}\right]>0.$$

This shows that, if the lower amplitude envelope of a valid bucket is already higher than the upper envelope of its local limit, a global risk-positive result can be given at once. This criterion is sufficient and conservative.

It should be emphasized that this condition is only a sufficient early-stop rule for a risk-positive decision. If a bucket does not satisfy this lower-envelope crossing condition, it is not directly certified as risk-negative. It is either certified by the upper-envelope condition below or kept in the uncertain set $\Omega_{\mathrm{unc}}$ for local refinement and sequential verification. Therefore, the sufficient early-stop rule cannot by itself cause a missed risk-positive decision; it only determines whether the decision can be made before refinement.

Similarly, if, for a valid bucket *b*,

(27) $$\frac{\left|Y[b]\right|}{\underset{a\in A}{\inf}\left|G\left[-a\right]\right|}\leq \underset{k\in S_{b}^{\Psi }}{\inf}L_{\gamma ,\sigma }\left[k\right],$$

then the bucket makes no positive contribution to the global worst-case margin m(X). Because the risk variable m(X) in this work is defined by the pointwise amplitude margin, the decision object is the maximum exceedance of each discrete frequency bin relative to its mapped limit rather than the accumulated energy across multiple frequency bins or the aggregated reading after receiver detection. Therefore, several frequency bins that are individually below their mapped limits do not form an additional cumulative exceedance event in this model. This is a local result. It means that the dominant candidate component in bucket *b* can be removed with risk-negative certification as a possible risk-positive source. It does not prove global risk-negative by itself. Global risk-negative still needs all valid buckets related to the decision to be certified as risk-negative. The remaining buckets can also be checked later and removed from the risk-positive set after refinement.

Risk-positive certification and risk-negative certification are not logically symmetric. A risk-positive decision is an existential statement. One bucket with clear exceedance evidence is enough to give a global risk-positive decision. A risk-negative decision is a universal statement. All relevant buckets must be certified as risk-negative. This logical asymmetry gives the proposed method an early-stop advantage at this stage.

To avoid mixing the set partition in bucket-level certification with the early-stop flow in actual execution, let $\Omega_{\mathrm{chk}}$ denote the bucket set selected for checking during execution. The buckets certified as risk-positive form $\Omega_{{\mathrm{Risk}}^{+}}$. The buckets certified as risk-negative form $\Omega_{{\mathrm{Risk}}^{-}}$. The remaining buckets in $\Omega_{\mathrm{chk}}$ cannot be certified as risk-positive by the lower envelope crossing the upper limit envelope. They also cannot be removed as risk-negative by the upper envelope falling below the lower limit envelope. These buckets form the uncertain-bucket set $\Omega_{\mathrm{unc}}=\Omega_{\mathrm{chk}}\setminus \left(\Omega_{{\mathrm{Risk}}^{+}}\cup \Omega_{{\mathrm{Risk}}^{-}}\right)$.

This step further reduces the set that needs processing. The later high-cost in-bucket refinement is not applied to all buckets. It is only applied to the small number of buckets in $\Omega_{\mathrm{unc}}$.

### 3.4. Local Refinement for Uncertain Buckets

When $\Omega_{{\mathrm{Risk}}^{+}}=⌀$ and $\Omega_{\mathrm{unc}}\neq ⌀$, the uncertain buckets need local point decisions one by one. For the uncertain-bucket set $\Omega_{\mathrm{unc}}$, the bucket-level amplitude-envelope interval and the local limit-envelope interval are still not fully separated. They cannot give a clear risk decision yet. The uncertainty comes from the unknown in-bucket dominant offset $a_{b}$. The bucket-level interval comparison cannot yet become a single-frequency comparison. An additional circular shift *s* is introduced for this case. Since this paper uses $N=2^{m}$, *s* must be odd. Its inverse is denoted by $s^{-1}$.

Let $x_{\gamma ,\sigma }^{\left(s\right)}\left[n\right]$ be the circularly shifted version of the reordered time-domain sequence. Its frequency-domain representation is $X_{\gamma ,\sigma }^{\left(s\right)}\left[k\right]$. Apply the same flat-window filtering, folding, and *B*-point discrete Fourier transform as in Section 3.1 to $x_{\gamma ,\sigma }^{\left(s\right)}\left[n\right]$. This gives $Y_{s}\left[b\right]$. For $b\in \Omega_{\mathrm{unc}}$, the original bucket observation and the shifted bucket observation can be written as:

(28) $$Y\left[b\right]=X_{\gamma ,\sigma }\left[k_{b}^{*}\right]G\left[-a_{b}\right]+\varepsilon_{b},$$

(29) $$Y_{s}\left[b\right]=X_{\gamma ,\sigma }\left[k_{b}^{*}\right]W_{N}^{sk_{b}^{*}}G\left[-a_{b}\right]+\varepsilon_{b}^{\left(s\right)}$$

Here, $\varepsilon_{b}$ and $\varepsilon_{b}^{\left(s\right)}$ are the combined perturbation terms in the original observation and the shifted observation. Their ratio satisfies:

(30) $$R_{s}\left[b\right]≜\frac{Y_{s}\left[b\right]}{Y[b]}=W_{N}^{sk_{b}^{*}}\frac{1+\frac{\varepsilon_{b}^{\left(s\right)}}{X_{\gamma ,\sigma }\left[k_{b}^{*}\right]W_{N}^{sk_{b}^{*}}G\left[-a_{b}\right]}}{1+\frac{\varepsilon_{b}}{X_{\gamma ,\sigma }\left[k_{b}^{*}\right]G\left[-a_{b}\right]}}.$$

The fractional factor on the right-hand side is close to 1. It only differs from 1 by a higher-order small term. This gives the first-order approximation:

(31) $$R_{s}\left[b\right]\approx \exp\left(-j\frac{2\pi }{N}s\left(b\cdot \mathrm{STEP}+a_{b}\right)\right).$$

This shows that, for an uncertain bucket, the amplitude of Y[b] alone is not enough for bucket-level certification. The phase ratio $R_{s}\left[b\right]$ still keeps the modulo-*N* location information of the in-bucket dominant offset $a_{b}$. The offset estimate can then be solved as:

(32) $$\begin{array}{cc} {\hat{a}}_{b}= & \left(\left(\left(s^{-1}\cdot \mathrm{round}\left(-\frac{N}{2\pi }\arg\left(R_{s}\left[b\right]\right)\right)\right)\;\mathrm{mod}\;N\right)-b\cdot \mathrm{STEP}+\frac{\mathrm{STEP}}{2}\right)\\ \; & \;\mathrm{mod}\;\mathrm{STEP}-\frac{\mathrm{STEP}}{2}. \end{array}$$

Once ${\hat{a}}_{b}$ is recovered, the uncertainty in the original bucket-level interval comparison can be changed into a check on one candidate frequency bin. The recovered candidate index is defined as ${\hat{k}}_{b}^{*}≜b\cdot \mathrm{STEP}+{\hat{a}}_{b}$. If

(33) $$\frac{\left|Y[b]\right|}{\left|G[-{\hat{a}}_{b}]\right|}>L_{\gamma ,\sigma }\left[{\hat{k}}_{b}^{*}\right],$$

then the bucket still gives clear exceedance evidence after refinement. Otherwise, the bucket can be removed from the possible risk-positive sources. The size of $\Omega_{\mathrm{unc}}$ is usually much smaller than the size of $\Omega_{\mathrm{iso}}$. This refinement only needs to be used on a small number of unresolved buckets. The same high-cost location recovery does not need to be used on all buckets.

This paper also does not refine all uncertain buckets in the same way. The next section introduces a sequential worst-case verification strategy. Buckets in $\Omega_{\mathrm{unc}}$ are processed one by one according to their potential risk order. This further reduces the average computational cost.

### 3.5. Sequential Verification Strategy for Worst Exceedance Evidence

After the envelope comparison, if the uncertain set $\Omega_{\mathrm{unc}}$ is still nonempty, the remaining task becomes an ordering optimization problem. Berend et al. formulated the optimal ordering of independent tests with precedence constraints in 2014, where test outcomes may already determine the final decision before all tests are executed, and the ordering is selected to reduce the expected resource consumption [32]. Following this idea, this section investigates the order in which the uncertain buckets should be verified so that the early stopping advantage of the proposed method can be maximized.

To define a lower-bound-driven value directly related to bucket-level risk-positive evidence, the normalized worst-case indicator is defined as:

(34) $$s_{\mathrm{lb}}\left(b\right)≜\frac{\left[\underset{k\in S_{b}^{\Psi }}{\sup}L_{\gamma ,\sigma }\left[k\right]\right]\left[\underset{a\in A}{\sup}\left|G\left[-a\right]\right|\right]-\left|Y\left[b\right]\right|}{\left[\underset{a\in A}{\sup}\left|G\left[-a\right]\right|\right]\left(\underset{k\in S_{b}^{\Psi }}{\sup}L_{\gamma ,\sigma }\left[k\right]-\underset{k\in S_{b}^{\Psi }}{\inf}L_{\gamma ,\sigma }\left[k\right]\right)+\tau }.$$

Here, τ>0 is a very small positive number. It is used to avoid denominator degeneration. A smaller $s_{\mathrm{lb}}\left(b\right)$ means that the bucket should be verified earlier.

The buckets in $\Omega_{\mathrm{unc}}$ are sorted in ascending order of $s_{\mathrm{lb}}\left(b\right)$. The reordered sequence is denoted by $b^{\left(1\right)},b^{\left(2\right)},\ldots ,b^{\left(|\Omega_{\mathrm{unc}}|\right)}$. This is the sequential verification order used in this paper. Uncertain buckets closer to the risk-positive boundary under the bucket-level lower envelope are verified first. Buckets farther from the boundary are verified later.

Under this order, the local refinement process in Section 3.4 is used for the *i*-th bucket $b^{\left(i\right)}$. It recovers the candidate offset ${\hat{a}}_{b^{\left(i\right)}}$ and the candidate index ${\hat{k}}_{b^{\left(i\right)}}^{*}$. It also builds the local amplitude estimate ${\hat{A}}_{b^{\left(i\right)}}$ and the corresponding limit $L_{\gamma ,\sigma }\left[{\hat{k}}_{b^{\left(i\right)}}^{*}\right]$. The refined local margin is defined as $\hat{\Delta }\left(b^{\left(i\right)}\right)≜{\hat{A}}_{b^{\left(i\right)}}-L_{\gamma ,\sigma }\left[{\hat{k}}_{b^{\left(i\right)}}^{*}\right]$. The sequential stopping time is then defined as:

(35) $$T≜\inf\left\{i\in \left\{1,2,\ldots ,\left|\Omega_{\mathrm{unc}}\right|\right\}\;|\;\hat{\Delta }\left(b^{\left(i\right)}\right)>0\right\},$$

If the above set is empty, set $T=|\Omega_{\mathrm{unc}}|$. During the sequential process, if any bucket is still above the limit after refinement, global risk-positive can be given. Global risk-negative can be given only after all buckets in $\Omega_{\mathrm{unc}}$ are checked and removed as risk-negative.

### 3.6. Method Summary

The proposed method works as follows. Randomized spectral reordering, flat-window local aggregation, and folded sampling first change the original length-*N* frequency-domain analysis problem into *B* bucket-level observations. The method then checks the relative positions of the bucket-level amplitude envelope and the local limit envelope. Most clearly risk-negative or clearly risk-positive buckets can be removed at this step. A small number of uncertain buckets remain in the gray zone. For these buckets, circular-shift observations and phase-ratio recovery are used to refine the dominant in-bucket offset. The bucket-level interval comparison then becomes a single-frequency comparison. The lower-bound-driven sequential worst-case verification strategy is used at the end. It exposes possible risk-positive evidence first.

**Figure 2:**
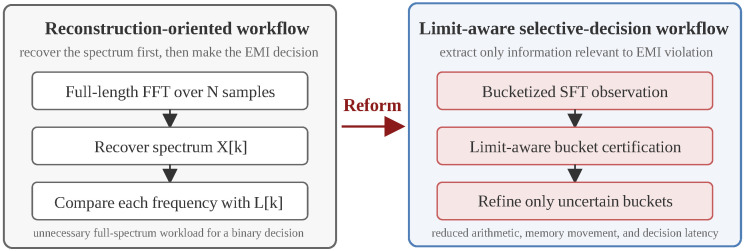
Paradigm shift from spectrum reconstruction to selective decision.

As shown in Figure 2, the proposed method changes the basic flow from full-spectrum recovery to task-tailored decision making. It avoids using FFT computation on many inactive frequency bins. These bins do not affect the final decision.

The overall procedure can be summarized by Algorithm 1:
**Algorithm 1** Proposed limit-aware selective decision method**procedure** HashToBins(x,γ,σ,g,B)      Compute $x_{\gamma ,\sigma }\left[n\right]=x\left[\left(\gamma n\right)\;\mathrm{mod}\;N\right]\cdot W_{N}^{\gamma \sigma n}$.      Compute $z\left[n\right]=g\left[n\right]\cdot x_{\gamma ,\sigma }\left[n\right]$.      Compute $\tilde{z}\left[r\right]=\sum_{q=0}^{N/B-1}z\left[r+qB\right]$.      Compute the *B*-point DFT of $\tilde{z}\left[r\right]$ and obtain Y[b].      **return** *Y* **end procedure** **procedure** PhaseRefine($Y,Y_{s},b,s$)      $R_{s}\left[b\right]\leftarrow Y_{s}\left[b\right]/Y\left[b\right]$,      $\phi_{s}\left(b\right)\leftarrow \arg\left(R_{s}\left[b\right]\right)$.      ${\hat{k}}_{b}\leftarrow \left(s^{-1}\cdot \mathrm{round}\left(-\frac{N\phi_{s}\left(b\right)}{2\pi }\right)\right)\;\mathrm{mod}\;N$.      ${\hat{a}}_{b}\leftarrow \left(({\hat{k}}_{b}-b\cdot \mathrm{STEP}+\mathrm{STEP}/2)\;\mathrm{mod}\;\mathrm{STEP}\right)-\mathrm{STEP}/2$.      ${\hat{k}}_{b}^{*}\leftarrow \left(b\cdot \mathrm{STEP}+{\hat{a}}_{b}\right)\;\mathrm{mod}\;N$.      **return** $\left({\hat{a}}_{b},{\hat{k}}_{b}^{*}\right)$ **end procedure** **procedure** LimitAwareEMICheck(x,L,γ,σ,g,B,s,τ)      Assume gcd(γ,N)=gcd(s,N)=1.      Y←HashToBins(x,γ,σ,g,B).      $G\leftarrow {\mathrm{DFT}}_{N}\left(g\right)$,      $\eta \leftarrow \gamma^{-1}\;\mathrm{mod}\;N$,      STEP←N/B.      A←[−STEP/2,STEP/2)∩Z,      $\Omega_{\mathrm{unc}}\leftarrow ⌀$.      $G_{\max}\leftarrow \max_{a\in A}\left|G\left[-a\right]\right|$,      $G_{\min}\leftarrow \min_{a\in A}\left|G\left[-a\right]\right|$.      Compute $L_{\gamma ,\sigma }\left[k\right]=L\left[\left(\eta k+\sigma \right)\;\mathrm{mod}\;N\right]$.      **for** b∈{0,1,…,B−1} **do**            $S_{b}^{\Psi }\leftarrow \left\{\left(b\cdot \mathrm{STEP}+a\right)\;\mathrm{mod}\;N:a\in A\right\}\cap \Psi_{\gamma ,\sigma }$.            **if** $S_{b}^{\Psi }=⌀$ **then**                  **continue**            **end if**            ${\mathrm{amp}}_{\mathrm{low}}\left(b\right)\leftarrow \left|Y\left[b\right]\right|/G_{\max}$.            ${\mathrm{amp}}_{\mathrm{high}}\left(b\right)\leftarrow \left|Y\left[b\right]\right|/G_{\min}$.            $L_{\min}\left(b\right)\leftarrow \min_{k\in S_{b}^{\Psi }}L_{\gamma ,\sigma }\left[k\right]$.            $L_{\max}\left(b\right)\leftarrow \max_{k\in S_{b}^{\Psi }}L_{\gamma ,\sigma }\left[k\right]$.            **if** ${\mathrm{amp}}_{\mathrm{low}}\left(b\right)>L_{\max}\left(b\right)$ **then**                  **return** risk-positive            **else if** ${\mathrm{amp}}_{\mathrm{high}}\left(b\right)\leq L_{\min}\left(b\right)$ **then**                  **continue**            **else**                  $\Omega_{\mathrm{unc}}\leftarrow \Omega_{\mathrm{unc}}\cup \left\{b\right\}$.            **end if**      **end for**      **if** $\Omega_{\mathrm{unc}}=⌀$ **then**            **return** risk-negative      **end if**      Let $x_{\gamma ,\sigma }^{\left(s\right)}\left[n\right]=x_{\gamma ,\sigma }\left[\left(n-s\right)\;\mathrm{mod}\;N\right]$.      $Y_{s}\leftarrow \mathrm{HashToBins}\left(x_{\gamma ,\sigma }^{\left(s\right)},1,0,g,B\right)$.      **for** $b\in \Omega_{\mathrm{unc}}$ **do**            $s_{\mathrm{lb}}\left(b\right)\leftarrow \frac{L_{\max}\left(b\right)-{\mathrm{amp}}_{\mathrm{low}}\left(b\right)}{L_{\max}\left(b\right)-L_{\min}\left(b\right)+\tau }$.      **end for**      Sort $\Omega_{\mathrm{unc}}$ in ascending order of $s_{\mathrm{lb}}\left(b\right)$ and obtain $b^{\left(1\right)},b^{\left(2\right)},\ldots ,b^{\left(|\Omega_{\mathrm{unc}}|\right)}$.      **for** i=1 **to** $|\Omega_{\mathrm{unc}}|$ **do**            $\left({\hat{a}}_{b^{\left(i\right)}},{\hat{k}}_{b^{\left(i\right)}}^{*}\right)\leftarrow \mathrm{PhaseRefine}\left(Y,Y_{s},b^{\left(i\right)},s\right)$.            ${\hat{A}}_{b^{\left(i\right)}}^{\left(\mathrm{pt}\right)}\leftarrow |Y\left[b^{\left(i\right)}\right]|/|G\left[-{\hat{a}}_{b^{\left(i\right)}}\right]|$.            **if** ${\hat{A}}_{b^{\left(i\right)}}^{\left(\mathrm{pt}\right)}>L_{\gamma ,\sigma }\left[{\hat{k}}_{b^{\left(i\right)}}^{*}\right]$ **then**                  **return** risk-positive            **end if**      **end for**      **return** risk-negative **end procedure**

## 4. Validation and Engineering Applicability Analysis

### 4.1. Monte Carlo Consistency Verification Against the Full-Spectrum Baseline

#### 4.1.1. Experimental Design

To check whether the proposed method gives the same decision results as the full-spectrum baseline under ideal sparse spectra, this paper generates random sparse spectral samples with a Monte Carlo procedure. The output of the proposed method is compared with the full-spectrum baseline labels. These labels are obtained by full-FFT pointwise limit comparison. The fixed parameters are: sampling frequency $F_{s}=2.5\;\mathrm{GHz}$, number of samples $N=2^{20}$, number of buckets B=8192, and STEP=N/B=128. The total frequency-domain sparsity is K=64. This value is used by default unless otherwise stated.

A total of 10,000 ideal sparse spectral samples are generated. Among them, 5000 samples are reference risk-negative. The other 5000 samples are reference risk-positive. For each sample, a dominant support $\Lambda_{K}$ of size *K* is randomly selected in the band of interest Ψ. The tail spectrum is set to zero or to a very weak perturbation. It is much smaller than the dominant spectrum. Thus, $\nu_{b}\approx 0$ holds. This section uses large-scale ideal random inputs to check the algorithm logic under completely sparse spectra.

#### 4.1.2. Results and Analysis

To avoid ambiguity, a reference risk-negative sample predicted as risk-negative, a reference risk-negative sample predicted as risk-positive, a reference risk-positive sample predicted as risk-negative, and a reference risk-positive sample predicted as risk-positive are denoted as true negative (TN), false positive (FP), false negative (FN), and true positive (TP), respectively. The false positive rate (FPR) and false negative rate (FNR) are defined as:

(36) $$FPR=\frac{FP}{FP+TN},\;\;\;\;\;\;FNR=\frac{FN}{FN+TP}.$$

For the 10,000 random samples that satisfy the ideal sparsity condition, the reference labels from the full-spectrum pointwise reference X[k] and the limit sequence L[k] are the same as the results of the proposed method. The overall decision consistency is 100%. FPR and FNR are both 0. The 100% result here is mainly used to verify that the proposed flow does not introduce additional logical decision bias. It does not mean that the proposed algorithm can maintain 100% decision consistency with the full-spectrum pointwise reference under all conditions. The degradation cases will be discussed in detail in Section 4.2.

**Figure 3:**
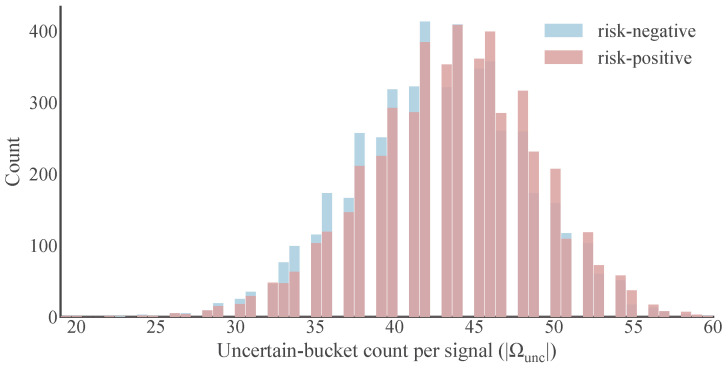
Distribution of the number of uncertain buckets $|\Omega_{\mathrm{unc}}|$.

From the bucket-level screening behavior, the proposed method first screens buckets by the envelope comparison in Section 3.3. Figure 3 shows the number of buckets entering the uncertain set $\Omega_{\mathrm{unc}}$ after bucket-level screening. The values of $|\Omega_{\mathrm{unc}}|$ are clearly concentrated across samples. This shows that, under ideal sparse spectra, many valid buckets can be directly certified by the relative position of the amplitude-envelope interval $A_{b}$ and the local-limit-envelope interval $L_{b}$. They do not need to enter the refinement stage in Section 3.4.

**Figure 4:**
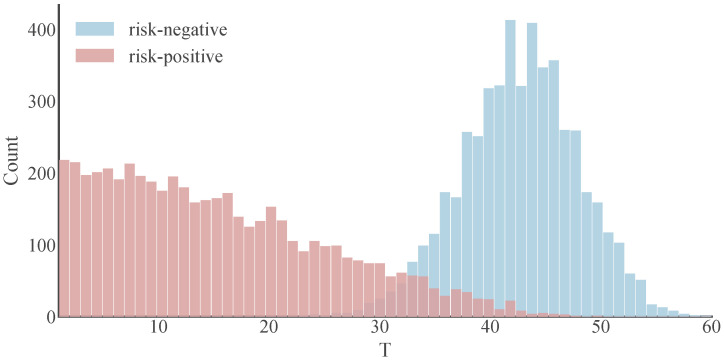
Distribution of the number of buckets *T* actually entering decoding.

Figure 4 shows the distribution of the number of buckets *T* that actually enter decoding for each signal. The mean value is about 28.82. This is much smaller than the spectral sparsity K=64. Even for samples that need further refinement, only a small number of buckets need phase recovery and single-frequency comparison. This number is far smaller than the number of all dominant spectral lines.

### 4.2. Performance Degradation Outside the Applicable Conditions

#### 4.2.1. Experimental Design

Unlike the random sparse spectral samples in Section 4.1, this subsection builds simulated spectral samples. It uses these samples to examine the applicable boundary of the method.

Previous studies show that EMI in power electronic converters is shaped by device switching and parasitic networks together. From the view of third-generation semiconductor power converters, Wu et al. pointed out that high dv/dt, high switching frequency, and parasitic ringing can broaden the EMI source spectrum. They can also form conducted EMI through common-mode and differential-mode paths [33]. Real EMI spectra have clear structured features. They are not uniformly random. In switching power supplies, conducted-noise emission is often concentrated around the switching frequency and its harmonics. Frequency modulation can spread each switching-frequency harmonic into sideband harmonics. Ringing-related emissions need separate treatment, such as snubbers or soft diodes [34]. More generally, EMI signals can be seen as a mixture of broadband/transient components and narrowband/continuous-wave components. Decomposition helps to explain the interference-generation mechanism [13]. In the engineering perturbation-spectrum construction in this section, $X_{K}$ and $R_{K}$ are further refined. The dominant spectrum $X_{K}$ is written as:

(37) $$X_{K}\left[k\right]=\sum_{m\in H}A_{m}e^{j\phi_{m}}\delta \left[k-k_{m}\right]+\sum_{q=1}^{Q_{d}}B_{q}e^{j\phi_{q}}\delta \left[k-k_{q}^{\left(d\right)}\right].$$

Here, H is the set of visible harmonics in the band of interest Ψ. $k_{m}$ corresponds to the switching fundamental frequency and its harmonic locations. $A_{m}$ denotes the dominant harmonic amplitude. In the second term, $k_{q}^{\left(d\right)}$ and $B_{q}$ denote a small number of local narrowband parasitic peaks. Their amplitudes are high enough to enter the best *K*-term support. This is decided by their relative amplitudes and the best *K*-term truncation. It is not decided only by their physical source. This section focuses on the physical meaning of the dominant support $\Lambda_{K}$ in Section 3.1.

The corresponding tail perturbation spectrum $R_{K}$ is written as

(38) $$R_{K}\left[k\right]=R_{sb}\left[k\right]+R_{res}\left[k\right]+R_{bg}\left[k\right].$$

where

(39) $$R_{sb}\left[k\right]=\sum_{m\in H}\sum_{l=1}^{L_{m}}a_{m,l}e^{j\phi_{m,l}}\delta \left[k-\left(k_{m}+\Delta k_{m,l}\right)\right].$$

denotes the weak sideband terms around the main harmonics. It describes spectral skirts caused by spread-spectrum modulation [35], intermodulation sidebands, clock jitter, or periodic small perturbations that commonly appear in closed-loop EMI control systems. Further,

(40) $$R_{res}\left[k\right]=\sum_{q=1}^{Q_{w}}b_{q}e^{j\psi_{q}}\;h_{q}\left(k-k_{q}^{\left(c\right)}\right)$$

denotes weak parasitic resonance peak clusters that do not enter the dominant support. Here, $h_{q}\left(\cdot \right)$ is a finite-width local peak envelope. It describes narrowband clustered structures introduced by package parasitics, power-loop ringing, reverse recovery, and PCB/cable resonances. This modeling choice is consistent with experimental EMI studies on power converters, where resonant peaks and localized time–frequency components have been linked to the above noise-generation mechanisms [22]. Further,

(41) $$R_{bg}\left[k\right]=\beta \left[k\right]e^{j\theta_{k}}.$$

denotes continuous thermal noise and control noise, where β[k] is a low-amplitude envelope that varies slowly with frequency [13]. Thus, the tail term $R_{K}$ in Section 3.1 is further decomposed in this section into three physically interpretable non-dominant components.

To study how the proposed method degrades outside its applicable conditions, this section starts from the mother spectra above. It gradually strengthens their non-dominant tail components. This makes the earlier small-perturbation assumption become invalid step by step.

This section does not directly add artificial amplification to $\varepsilon_{b}$ or $\nu_{b}$. $\nu_{b}$ is a bucket-domain quantity. Its value is not decided by only one type of perturbation. For the same original spectrum, different hashing states can give different in-bucket competition and different out-of-bucket leakage. If the terms of $\nu_{b}$ are directly made worse in analysis, the construction would leave the physical generation process of the original spectrum. It would become a non-engineering construction. The error would be set first in the bucket domain. The sample would then be inferred backward. This paper still defines the degradation mechanism from the modeled tail spectrum $R_{K}\left[k\right]$. In this way, the growth of $\nu_{b}$ is naturally caused by spectral-shape changes.

For sample organization, this section first generates 1000 mother spectra by default. Risk-negative and risk-positive samples have the same number. For each degradation configuration, 500 samples are randomly selected for evaluation. The same mother-spectrum pool is used under different degradation strengths. This avoids changes in the mother-spectrum distribution hiding the effect of the degradation strength itself. The reference labels in each degradation configuration are recalculated from the degraded spectra by full-FFT pointwise limit comparison.

**Figure 5:**
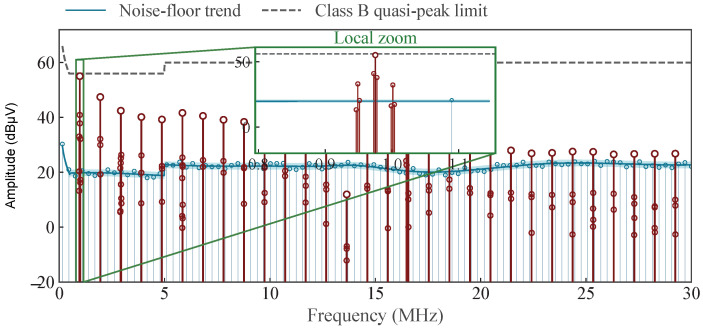
Representative risk-negative spectrum under the mapped reference limit. Cyan markers and stems denote background-floor spectral samples, dark-red markers and stems denote non-background spectral components, and darker-red stems highlight harmonic centers. The cyan curve denotes the noise-floor trend, the dashed black curve denotes the Class B quasi-peak limit, and the green inset provides a local zoom of the limit-critical region.

**Figure 6:**
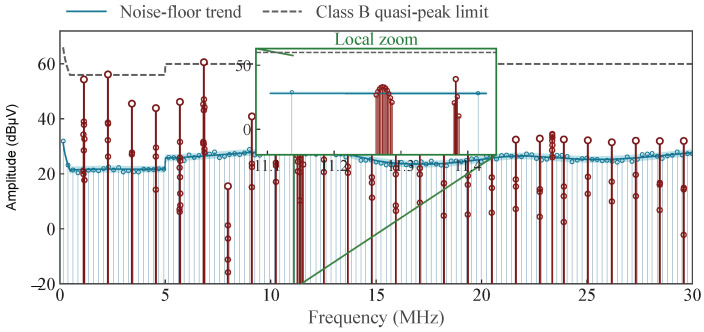
Representative risk-positive spectrum under the mapped reference limit. Cyan markers and stems denote background-floor spectral samples, dark-red markers and stems denote non-background spectral components, and darker-red stems highlight harmonic centers. The cyan curve denotes the noise-floor trend, the dashed black curve denotes the Class B quasi-peak limit, and the green inset provides a local zoom of the limit-critical region.

Figure 5 and Figure 6 show representative spectra of the risk-negative and risk-positive samples constructed in this section.

The paper then defines three degradation families. Each family has a degradation-strength index. Let the three tail templates of a mother spectrum be $R_{sb}^{\left(0\right)}\left[k\right]$, $R_{res}^{\left(0\right)}\left[k\right]$, and $R_{bg}^{\left(0\right)}\left[k\right]$. For any mother sample, the dominant part $X_{K}\left[k\right]$ is kept fixed. Only one type of tail template is amplified as a whole. This produces a sample family along a single degradation direction.

The first family is the continuous-background enhancement family. In this family, only the continuous low-amplitude background term $R_{bg}^{\left(0\right)}$ is amplified. $R_{sb}^{\left(0\right)}$ and $R_{res}^{\left(0\right)}$ stay at their mother-spectrum levels. Its degradation strength is defined as $\rho_{bg}≜\|R_{bg}{\|}_{2}/{\left\|X_{K}\right\|}_{2}$. The second family is the main-harmonic sideband enhancement family. In this family, only the weak sideband term $R_{sb}^{\left(0\right)}\left[k\right]$ is amplified. Its degradation strength is defined as $\rho_{sb}≜\|R_{sb}{\|}_{2}/{\left\|X_{K}\right\|}_{2}$. The third family is the weak parasitic-peak-cluster enhancement family. In this family, only the parasitic peak-cluster term $R_{res}^{\left(0\right)}\left[k\right]$ outside the dominant support is amplified. Its degradation strength is defined as $\rho_{res}≜\|R_{res}{\|}_{2}/{\left\|X_{K}\right\|}_{2}$.

Based on these definitions, the three degradation families are extended separately along $R_{bg}$, $R_{sb}$, and $R_{res}$. The goal is to see which part of the proposed method degrades first when one component keeps increasing. It also checks whether different degradation mechanisms have the same performance turning point. This helps to clarify the applicable range of the method.

To quantify the bucket-collision structure caused by practical non-dominant spectral components, this subsection defines the multi-peak bucket ratio before the result analysis. Since $\Lambda_{K}$ has been defined as the inband best-*K* approximation support, this subsection further uses $\Lambda_{\mathrm{eff}}$ to denote the degraded best-*K* approximation support and $\Lambda_{\mathrm{eff}}^{\gamma ,\sigma }$ to denote the reordered effective dominant support after randomized spectral reordering, with $|\Lambda_{\mathrm{eff}}^{\gamma ,\sigma }|=K_{\mathrm{eff}}$. For the checked bucket set $\Omega_{\mathrm{chk}}$, the multi-peak bucket ratio is defined as

(42) $$r_{\mathrm{mp}}=\frac{1}{|\Omega_{\mathrm{chk}}|}\sum_{b\in \Omega_{\mathrm{chk}}}1\left\{\left|\Lambda_{\mathrm{eff}}^{\gamma ,\sigma }\cap S_{b}^{\Psi }\right|\geq 2\right\}.$$

Here, 1{·} is the indicator function. Thus, $r_{\mathrm{mp}}$ measures the fraction of checked buckets that contain two or more effective dominant frequency components after randomized reordering.

#### 4.2.2. Result Analysis

**Figure 7:**
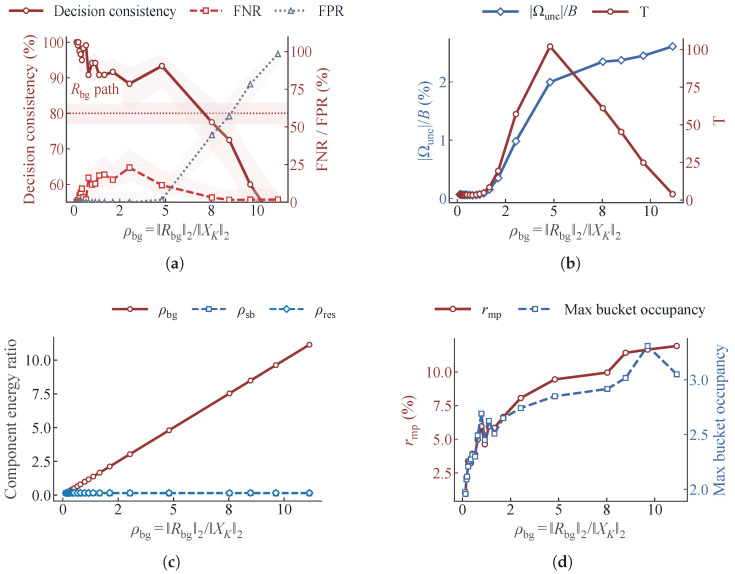
Decision-consistency degradation and bucket-structure evolution under the continuous-background enhancement path; (**a**) decision consistency, FNR, and FPR; (**b**) uncertain-bucket ratio and decoded-bucket count; (**c**) component energy ratios; (**d**) multi-peak bucket ratio and maximum bucket occupancy.

The continuous-background enhancement path is shown in Figure 7. This path changes along $\rho_{bg}$. It raises the low-amplitude background over the whole band. When $\rho_{bg}$ increases from 0.1355 to 3.03, decision consistency drops from 100% to 88.33%. FPR stays at 0. FNR rises to 22.95%. This shows that early background perturbation mainly weakens the lower-bound evidence of reference risk-positive buckets. Some risk-positive samples stay in the gray zone. When $\rho_{bg}$ further increases to about 4.80, $|\Omega_{\mathrm{unc}}|/B$ rises to about 2.0%. The number of decoded buckets reaches a peak of 102. The multi-peak ratio increases from about 1% to more than 9%. This shows that many weak components enter the bucket-level observations. They increase the uncertainty of the amplitude envelopes. At $\rho_{bg}=8.48$, FPR rises to 57.14%. This means that the strong background uplift moves some mother risk-negative samples toward a degraded risk-positive state. The main degradation mechanism of the continuous-background path is the dilution of global sparsity and the weakening of bucket-level envelope separation.

**Figure 8:**
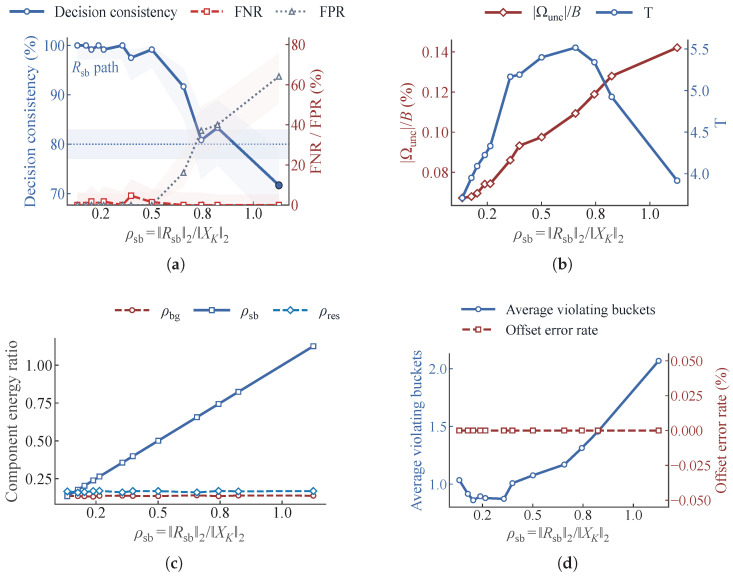
Decision-consistency degradation and local decoding behavior under the main-harmonic sideband enhancement path; (**a**) decision consistency, FNR, and FPR; (**b**) uncertain-bucket ratio and decoded-bucket count; (**c**) component energy ratios; (**d**) average risk-positive buckets and offset decoding error.

The main-harmonic sideband enhancement path is shown in Figure 8. This path changes along $\rho_{sb}$. It shows degradation the earliest. At $\rho_{sb}=0.3986$, decision consistency is still 97.50%. When $\rho_{sb}$ increases to 0.6566, FPR rises to 16.13%. At $\rho_{sb}=1.1257$, FPR reaches 64.15%. During this process, the multi-peak ratio stays at about 1%. The offset error remains 0. This shows that the degradation mainly happens in the bucket-level exclusion stage. Sideband energy is added directly around the main harmonics. These locations are usually closest to the limit boundary. Even a small sideband enhancement can raise the amplitude envelope of key buckets. It can quickly reduce the local separation between risk-negative and risk-positive buckets. This explains why $\rho_{sb}$ is the most sensitive of the three paths.

**Figure 9:**
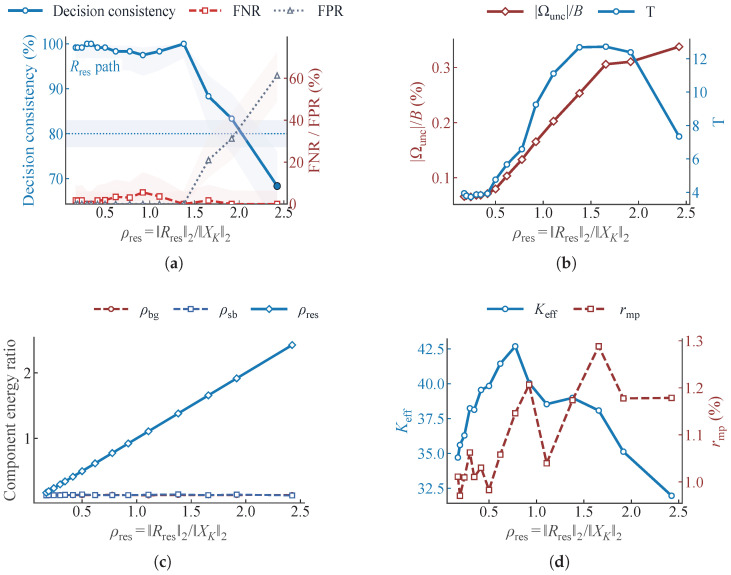
Decision-consistency degradation and effective-dominant-structure reorganization under the parasitic-resonance enhancement path; (**a**) decision consistency, FNR, and FPR; (**b**) uncertain-bucket ratio and decoded-bucket count; (**c**) component energy ratios; (**d**) effective dominant support size and multi-peak bucket ratio.

The weak parasitic-peak-cluster enhancement path is shown in Figure 9. This path changes along $\rho_{res}$. It is more stable in the early region. When $\rho_{res}$ increases from 0.1714 to 1.3803, decision consistency stays close to or at 100%. The turning point appears around $\rho_{res}=1.6567$. Decision consistency drops to 88.33%. FPR rises to 20.97%. At $\rho_{res}=2.4248$, FPR rises to 61.29%. Along this path, the multi-peak ratio stays around 1%. The offset error also stays at 0. This shows that the degradation mainly comes from changes in the effective dominant structure. $K_{\mathrm{eff}}$ first rises from about 34.7 to 42.7. It then falls back to about 32. This means that some local narrowband peaks in the tail gradually enter the effective dominant set under fixed-*K* truncation. The dominant support is then reordered internally. The main effect of the parasitic-peak-cluster path is to weaken the dominance of the original main-harmonic skeleton. It does not create a large number of bucket collisions.

Overall, the three degradation types damage the boundary in three different ways. Continuous-background enhancement weakens sparsity through fullband weak components. Main-harmonic sideband enhancement reduces the envelope separation in limit-sensitive regions. Parasitic-peak-cluster enhancement changes the effective dominant support under fixed-*K* truncation.

### 4.3. Applicable Range and Engineering Boundary

Section 4.2 studies the continuous-background, main-harmonic sideband, and parasitic-peak-cluster directions separately. It only shows which mechanism makes the algorithm start to degrade. In real EMI spectra, non-dominant structures usually do not change along only one coordinate axis. They often drift together across several perturbation components. The sensing-chain-acquired spectral samples need to be mapped into the joint phase space of the three perturbation components. They also need to be compared directly with the performance boundary from a three-dimensional scan. This follows the computer-experiment approach. In this view, a complex algorithm or system is treated as a mapping from an input-parameter space to output responses. Structured sampling of this space can build empirical response surfaces. It can also identify stable operating regions [36].

For this purpose, this paper uses two groups of EMI data measured from a laboratory GaN switching power converter through the in situ EMI sensing chain reported by Chen et al. [4]. These data are calibrated into amplitude-equivalent frequency-domain observations and used as practical test samples. One group uses a fixed switching frequency without RSSM. It is denoted as no-RSSM. The other group uses the spectrum after RSSM is applied. It is denoted as RSSM. These two data groups are used because RSSM in the target on-chip closed-loop regulation system changes spectral-line broadening and neighboring sideband structure through spread-spectrum technology. Studying them is representative of the practical use of the method. Periodic spread-spectrum modulation has also been studied for EMI reduction in SiC motor-controller and inverter systems. Secondary frequency modulation can further reduce conducted-EMI peaks. This can work even within a limited spreading range [37].

To give the sensing-chain-acquired samples a unified and reproducible position in the three-perturbation phase space, the no-RSSM and RSSM spectra are processed by the same joint soft-decomposition rule. Let $A_{\mathrm{sense}}\left(f\right)$ be the linear-amplitude sequence of the spectrum acquired through the in situ EMI sensing chain in the band of interest. First, the no-RSSM spectrum is used as the reference steady-state spectrum. A comb-matching step is applied to its smoothed excess spectrum. This step estimates the base spacing $f_{bb}$ of the dominant harmonic skeleton in Section 4.2. It also determines the dominant harmonic locations $k_{m}$ and their frequency locations for m∈H. The no-RSSM and RSSM spectra are then both decomposed with this fixed set of dominant harmonic locations as the reference skeleton.

After the reference dominant harmonic skeleton is obtained, asymmetric least-squares smoothing is used in the linear-amplitude domain to extract the continuous background term $A_{bg}\left(f\right)$. This step follows the baseline-correction idea of asymmetric least-squares smoothing, which estimates a slowly varying background while suppressing the influence of localized peaks [38]. The remaining amplitude after subtracting $A_{bg}\left(f\right)$ from $A_{\mathrm{sense}}\left(f\right)$ is treated as the part to be assigned. Three soft scores are then built from the distance between each frequency point and the nearest dominant harmonic location $k_{m}$. They also use the prior formed by the local excess amplitude. The first score is concentrated around the dominant harmonic locations. It represents the dominant skeleton in $X_{K}$ formed by the switching fundamental and its harmonics. The second score covers the main-harmonic neighborhood. It suppresses the peak center. It represents sidebands and spectral skirts around the main peaks. The third score uses the local-excess prior and a distance gate away from the dominant harmonic locations. It represents local narrowband parasitic peak clusters away from the dominant skeleton. The three scores are weighted by fixed weights and normalized. They soft-assign the residual amplitude after background subtraction to $A_{X_{K}}\left(f\right)$, $A_{sb}\left(f\right)$, and $A_{res}\left(f\right)$. This gives the approximate decomposition relation $A_{\mathrm{sense}}\left(f\right)\approx A_{bg}\left(f\right)+A_{X_{K}}\left(f\right)+A_{sb}\left(f\right)+A_{res}\left(f\right)$.

It should be noted that $A_{X_{K}}$, $A_{sb}$, $A_{res}$, and $A_{bg}$ do not form a strictly unique physical orthogonal decomposition. They are engineering descriptors. They describe the main directions and relative strengths of the deviation of a real spectrum from the dominant skeleton.

After these four components are obtained, this paper uses a discrete $L_{2}$ norm weighted by the frequency sampling interval to define the three perturbation coordinates. The spectrum acquired through the in situ EMI sensing chain is then mapped into the three-dimensional coordinates $\left(\rho_{bg},\rho_{sb},\rho_{res}\right)$ under the unified soft-decomposition rule.

**Figure 10:**
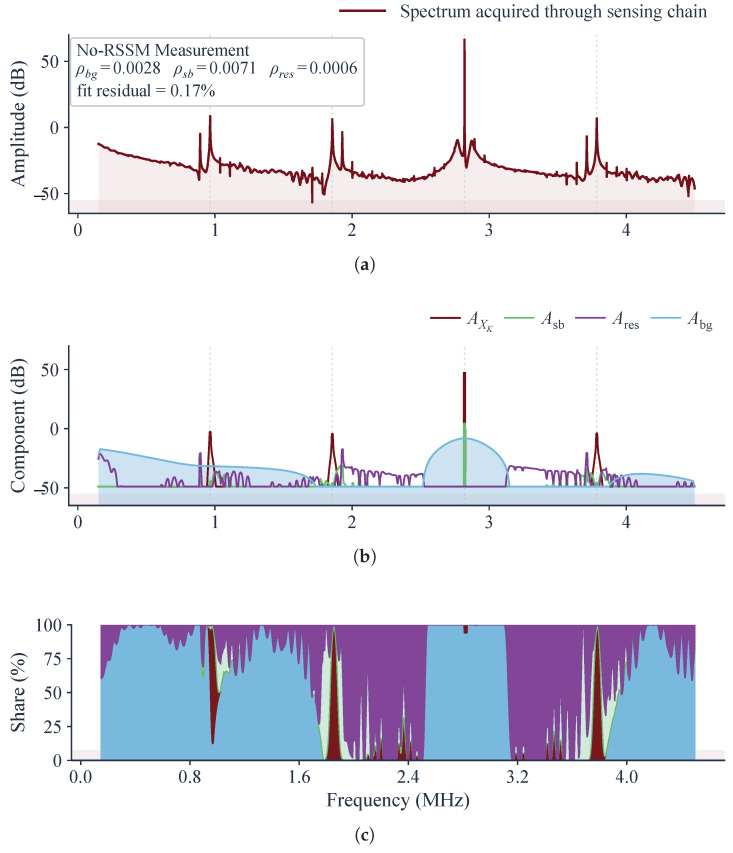
Joint soft decomposition of the spectrum acquired through the in situ EMI sensing chain under the no-RSSM condition; (**a**) sensing-chain-acquired spectrum and fitted perturbation coordinates, where the light-red shaded area fills the region between the measured spectrum and the plotting floor for visualization; (**b**) frequency-domain decomposition into dominant skeleton, sidebands, parasitic residuals, and background, where the vertical dotted guide lines indicate the reference harmonic-center locations; (**c**) relative component share across frequency, where the stacked colors follow the same component-color mapping as in (**b**).

**Figure 11:**
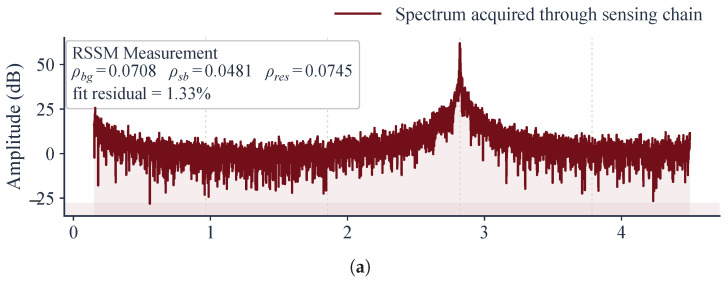

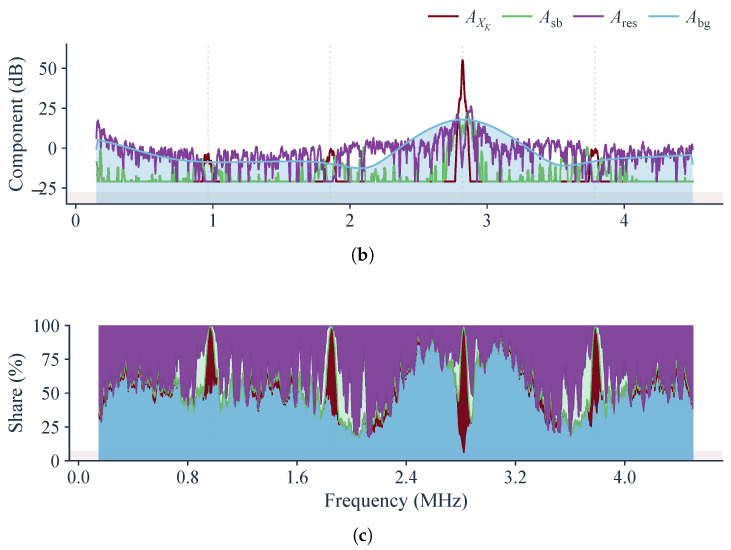
Joint soft decomposition of the spectrum acquired through the in situ EMI sensing chain under the RSSM condition; (**a**) sensing-chain-acquired spectrum and fitted perturbation coordinates, where the light-red shaded area fills the region between the measured spectrum and the plotting floor for visualization; (**b**) frequency-domain decomposition into dominant skeleton, sidebands, parasitic residuals, and background, where the vertical dotted guide lines indicate the reference harmonic-center locations; (**c**) relative component share across frequency, where the stacked colors follow the same component-color mapping as in (**b**).

Figure 10 and Figure 11 show the decomposition results for no-RSSM and RSSM. Figure 10a and Figure 11a show the spectrum acquired through the in situ EMI sensing chain. The fitting residual is only 0.17% for the no-RSSM sample and 1.33% for the RSSM sample. This shows that the decomposition fits the original spectrum acquired through the in situ EMI sensing chain well. For visualization, only 0–4 MHz is shown in these figures, while the coordinate calculation still uses the full 0.15–30 MHz band. Figure 10b and Figure 11b show the frequency-domain shapes of $A_{X_{K}}$, $A_{sb}$, $A_{res}$, and $A_{bg}$. Under no-RSSM, the dominant skeleton is the main part. The sidebands, parasitic peak clusters, and background floor are very weak. Under RSSM, the dominant skeleton locations stay stable. The background floor, local peak clusters, and skirts around the main peaks rise clearly. Figure 10c and Figure 11c give the relative share of the four components in each frequency band. This shows that the coordinate-extraction process is not decided by threshold classification at a few frequency points. It is decided by the overall allocation of spectral energy among the four structures over the whole band.

The two sensing-chain-acquired samples are therefore mapped as:

(43) $$\rho^{\left(no-RSSM\right)}=\left(\rho_{bg},\rho_{sb},\rho_{res}\right)=\left(0.0028,0.0071,0.0006\right),$$

(44) $$\rho^{\left(RSSM\right)}=\left(\rho_{bg},\rho_{sb},\rho_{res}\right)=\left(0.0708,0.0481,0.0745\right).$$

**Figure 12:**
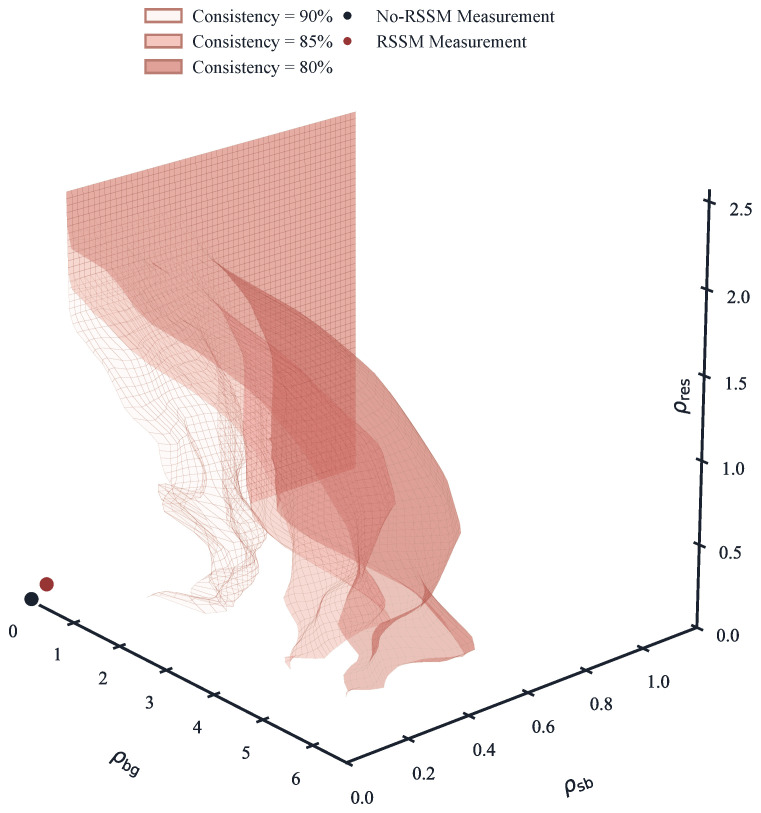
Decision consistency isosurfaces in the three-component perturbation phase space with projected measured samples.

**Figure 13:**
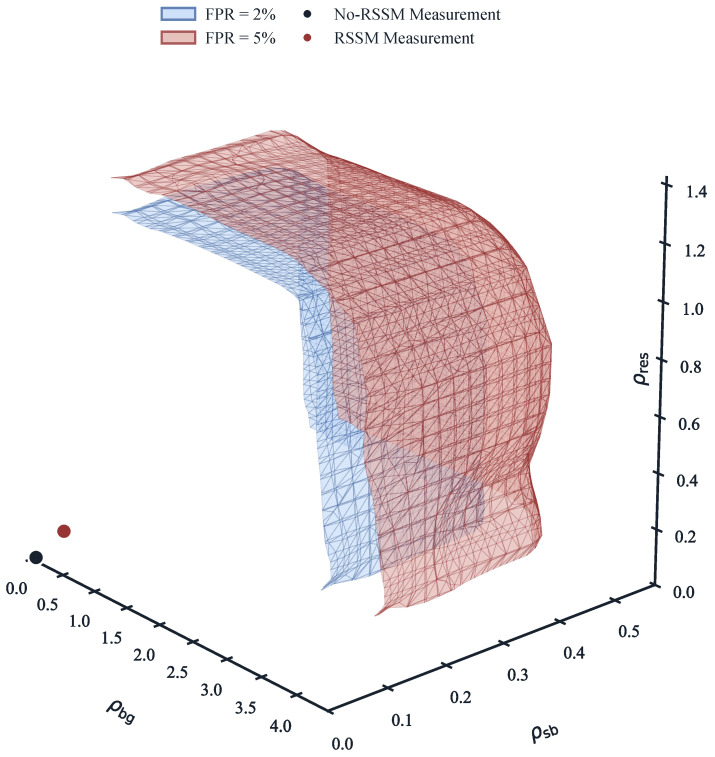
FPR isosurfaces in the three-component perturbation phase space with projected measured samples.

Based on this mapping, many simulated data are used to scan $\left(\rho_{bg},\rho_{sb},\rho_{res}\right)$ in the three-dimensional perturbation space. The decision consistency at each grid point is recorded as an empirical response surface. Let $\rho \equiv (\rho_{bg},\rho_{sb},\rho_{res})\in D$. The scan domain is $D=\left[\rho_{bg}^{-},\rho_{bg}^{+}\right]\times \left[\rho_{sb}^{-},\rho_{sb}^{+}\right]\times \left[\rho_{res}^{-},\rho_{res}^{+}\right]$. It is given by the actual experimental sampling range. The three-dimensional empirical performance functions ${\hat{C}}_{d}\left(\rho \right)$, $\hat{\mathrm{FPR}}\left(\rho \right)$, and $\hat{\mathrm{FNR}}\left(\rho \right)$ are then defined. They denote the average decision consistency, false positive rate, and false negative rate under a given joint perturbation configuration. Section 4.2.2 shows that the method first loses the ability to exclude risk-negative samples when it leaves the stable operating region. This section uses ${\hat{C}}_{d}\left(\rho \right)$ and $\hat{\mathrm{FPR}}\left(\rho \right)$ as the two main boundary measures. Figure 12 shows the isosurfaces of ${\hat{C}}_{d}\left(\rho \right)=90\%,85\%,80\%$. Figure 13 shows the isosurfaces of $\hat{\mathrm{FPR}}\left(\rho \right)=2\%$ and 5%. The two sensing-chain-acquired samples are also projected into these figures.

The empirical usable region is further defined as:

(45) $$R\left(C_{\min},\beta ,\eta \right)\equiv \left\{\rho \in D|{\hat{C}}_{d}\left(\rho \right)\geq C_{\min},\hat{\mathrm{FPR}}\left(\rho \right)\leq \beta ,\hat{\mathrm{FNR}}\left(\rho \right)\leq \eta \right\}.$$

Here, $C_{\min}$ is the minimum allowed decision consistency. β is the maximum allowed false positive rate. η is the maximum allowed false negative rate. For closed-loop regulation robustness, the engineering-realistic setting $C_{\min}=0.90$, β=0.02, and η=0.02 is used. Then R(0.90,0.02,0.02) is treated as the usable region of the proposed method. As indicated by the degradation results in Section 4.2.2, the FNR constraint is basically satisfied automatically in this low-perturbation operating region. The results in Figure 12 and Figure 13 show that the no-RSSM sample is near the low-ρ corner of the joint perturbation space. It is far from the 90% decision-consistency isosurface and the 2% FPR isosurface. It is clearly a stable operating point. The RSSM sample has shifted slightly compared with no-RSSM. Its projected location is still inside both types of isosurfaces. Its neighboring grid points still have 100% decision consistency, 0 FPR, and 0 FNR.

This result means that, for the GaN switching power supply EMI spectra studied in this paper, the measured operating points are still far from the three-dimensional decision-degradation boundary. The proposed method has good engineering usability for this real type of EMI spectrum. This spectrum is directly related to the reference closed-loop regulation system.

The empirical usable region does not mean that the algorithm can be used without limits over the whole parameter space. For boundary samples near $\partial R(C_{\min},\beta ,\eta )$, a more conservative engineering scheme is still needed. This is especially true for operating points close to the 90% decision-consistency isosurface or the 2% FPR isosurface. For example, a small negative bias can be added to the risk state variable m(X). The decision threshold can be shifted from 0 to −Δ. Here, Δ can be chosen as an amplitude margin of about 1 dB. The system then enters the suppression state earlier near the boundary.

## 5. Discussion and Performance Evaluation

### 5.1. Effectiveness of the Sequential Verification Strategy

#### 5.1.1. Modeling of Ordering Strategies and Worst-Case Construction

In Section 3.5, the uncertain-bucket set $\Omega_{\mathrm{unc}}$ is sorted by the lower-bound-driven metric $s_{\mathrm{lb}}\left(b\right)$. This gives a sequential verification order. Buckets that may contain the worst exceedance evidence are checked first. The choice of $s_{\mathrm{lb}}\left(b\right)$ still needs discussion.

To discuss this issue, this paper defines a unified family of ordering strategies. Besides the LB strategy, namely the lower-bound-driven strategy defined earlier, four comparison strategies are introduced.

First, the midpoint-driven strategy, MID, is defined. It is also called midpoint/mean-based. This strategy uses both the lower and upper bounds of the bucket-level amplitude. It uses their midpoint as the typical amplitude level of the dominant spectral line in the bucket. MID then sorts buckets by the distance between this midpoint and the upper edge of the local limit interval. MID represents local risk ordering in the mean sense. Compared with LB, it gives less weight to the worst case. It is closer to a compromise estimate of general risk.

Second, the upper-bound-driven strategy, UB, is defined. This strategy uses the upper end of the bucket-level amplitude envelope as the risk estimate of the dominant spectral-line amplitude. It checks how close this value is to the upper edge of the local limit interval. UB first processes the candidate buckets closest to the exceedance boundary under a pessimistic amplitude estimate. Compared with LB, UB is more sensitive to risk. It can cover risk-relevant buckets earlier. It can also move some interfering buckets to the front of the verification sequence. These buckets have high-amplitude upper bounds. They are certified as risk-negative after local refinement.

Third, an energy-driven strategy is introduced. It does not use any limit information. It is denoted as the |Y| strategy. Its ordering metric is defined as $s_{\left|Y\right|}\left(b\right)\equiv -\left|Y\left[b\right]\right|$. It only uses the observation strength itself. It does not use the closeness to the local limit. It is a task-agnostic heuristic ordering strategy.

Finally, random is defined as an uninformative baseline. It applies a uniform random ordering to $\Omega_{\mathrm{unc}}$. This strategy uses no risk-related statistic. It only represents the average stopping position and unfolding depth when no ordering information is available.

To make the differences among the ordering strategies more clear, this experiment builds a worst-case sequential-verification dataset. The dataset keeps only 1500 samples. These samples do not directly trigger risk-positive during envelope comparison. They should still be judged as risk-positive after local refinement. This makes the ordering strategy truly affect the sequential stopping time *T*. In each sample, only a very small number of buckets carry real risk-positive evidence. Usually, there is only one such bucket. Several near-threshold interfering buckets are placed among the other uncertain buckets. These interfering buckets are finally risk-negative. The target bucket is not clearly separated by bucket-level statistics. In this way, the decoding depth directly shows whether an ordering rule can find the true risk-positive evidence early.

#### 5.1.2. Effect of Ordering Strategies on Sequential Verification Efficiency

Using the worst-case sequential-verification dataset from Section 5.1.1, this paper compares the actual effect of five ordering strategies on risk-positive triggering efficiency. The bucket-level screening, local refinement, and final decision rules are fixed. Only the order of uncertain buckets in $\Omega_{\mathrm{unc}}$ is changed.

**Figure 14:**
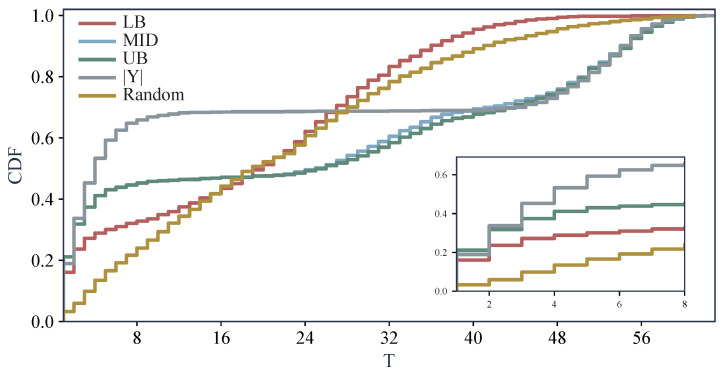
Empirical CDF comparison of risk-positive-triggering decode counts under different ordering strategies.

Figure 14 shows the empirical cumulative distribution functions of the risk-positive-triggering decode count *T* under different ordering strategies. No single strategy is best over the whole range. The curves cross in different decoding-depth regions. In the very small-*T* region, the |Y| curve rises fastest. It is most likely to trigger risk-positive within only a few steps. LB is less aggressive than |Y| at this early stage. As *T* increases, its CDF quickly becomes higher than the others. It stays ahead in the middle and later regions. This shows an advantage in average complexity and tail control. The MID and UB curves shift to the right. They usually need deeper decoding before true risk-positive evidence is found. Random is clearly worse in the early region. It performs better than MID and UB in the middle range. This shows that no risk information weakens early hits. Some biased ordering rules can still perform worse than the random baseline.

**Figure 15:**
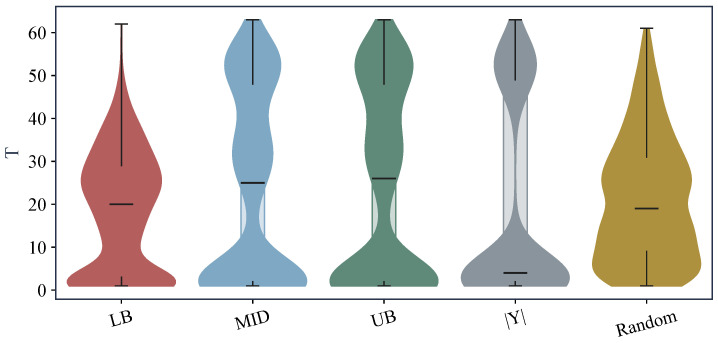
Distribution shape and tail behavior of risk-positive-triggering decode counts under different ordering strategies.

Figure 15 explains the degradation modes of the ordering metrics. The main part of the LB distribution is continuous. It mostly stays in the medium decoding-depth range. This shows that LB gives stable ordering across samples. The |Y| distribution is clearly split. Some samples have very low decode counts. Other samples form a long tail. This means that energy ordering depends strongly on whether the risk-positive bucket is also a high-energy bucket. If the high-energy bucket is only a near-threshold interferer, verification goes into a deeper scan. MID and UB both show a bimodal pattern with low and high positions. This shows that the interval midpoint or upper bound cannot always separate true risk-positive buckets from near-threshold risk-negative buckets. The random distribution has no clear main peak. The trigger position changes over a wide range. This matches the random unfolding behavior of an uninformative ordering. The advantage of LB comes from lower sample-to-sample variation and fewer ordering mistakes.

More detailed statistics are summarized in Table 2. LB has the lowest average risk-positive-triggering decode count, 18.45. It also has the lowest 90th percentile, 36. This shows its advantage in average complexity and worst-case control. The mean of |Y| is 18.53, almost the same as LB. Its median is only 4. P(T≤5) is 59.3%, much higher than the other strategies. This means that |Y| has the strongest early-triggering ability when the risk-positive bucket is clearly high-energy. The means of MID and UB rise to 23.98 and 24.40. Their 90th percentiles reach 54 and 55. Both are clearly worse than LB. Random has a mean of 21.05 and a 90th percentile of 41. Overall, it performs better than MID and UB.

Figure 14 and Figure 15, together with Table 2, give the following conclusion. If only the very early triggering ability in the best case is considered, |Y| is the strongest. If average complexity and tail worst-case control are considered, LB is better. This advantage fits the design goal of on-chip closed-loop EMI regulation better. In a closed-loop system, the key point is not an occasional very fast trigger for one sample. The key point is stable, predictable, and bounded average cost during continuous observation. The system also needs to expose the worst exceedance evidence reliably. For this reason, the later RTL implementation in this paper uses LB as the default ordering strategy.

### 5.2. RTL-Level Hardware Implementation, PPA Evaluation, and EMI Risk Feedback Interface

#### 5.2.1. Experimental Design and Functional Verification

To further evaluate hardware feasibility, this section maps the proposed method to an RTL structure. Synthesis, implementation, and PPA evaluation are completed on an FPGA platform.

The experiment uses Xilinx Vivado 2024.1 (AMD Xilinx, San Jose, CA, USA). All RTL designs are synthesized and implemented on a Xilinx Virtex UltraScale+ VU13P FPGA (AMD Xilinx, San Jose, CA, USA). The target device is xcvu13p-flga2577-3-e with speed grade −3. Considering the on-chip storage pressure in FPGA prototype verification, this section uses the hardware scale $N=2^{18}$. The sampling frequency follows the previous setting, $F_{s}=2.5\;\mathrm{GHz}$. The band of interest is still 0.15 MHz to 30 MHz. The input data use fixed-point representation. The limit table, bucket-level thresholds, and constants required for phase refinement are all stored on chip as lookup tables.

**Figure 16:**
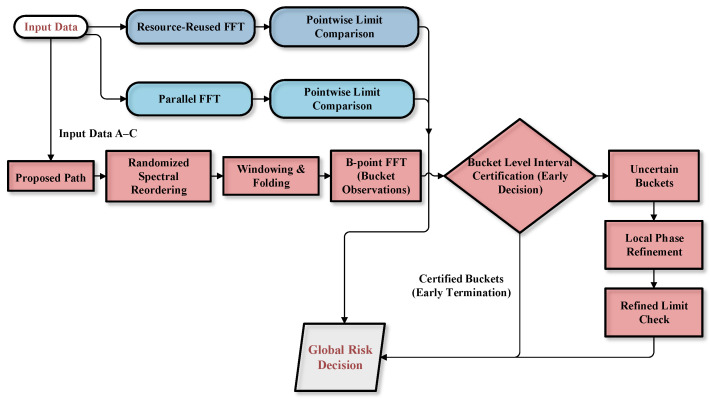
Data-flow comparison of the three hardware implementation routes.

For comparison, three hardware implementation routes are used. The first one is an area-first full-FFT baseline. It reduces arithmetic resources by highly reusing butterfly units. It is called the resource-reused FFT baseline. The second one is a throughput-first full-FFT baseline. It uses higher parallelism and double-buffered storage to reduce execution cycles. It is called the Parallel FFT Baseline. The third one is the proposed limit-aware implementation, called Proposed. All three designs are described in SystemVerilog. They use the same coding style, synthesis flow, and place-and-route flow. Their arithmetic precision is the same. All designs use a 10 ns clock period constraint. This corresponds to a target frequency of 100 MHz. Figure 16 shows the top-level data flows of the three implementations.

This paper uses self-implemented RTL designs with comparable optimization effort for all the compared schemes. The focus of the hardware comparison is to evaluate the architectural trade-off between the conventional flow of full-spectrum reconstruction followed by pointwise limit comparison and the proposed selective risk-decision flow rather than to compete with vendor-optimized FFT IP at the micro-architecture level. Vendor FFT IP is not used as the primary baseline because it represents a mature implementation with independent engineering optimization rather than a controlled algorithmic and architectural baseline. For example, the AMD/Xilinx FFT LogiCORE provides multiple architecture options, including pipelined streaming I/O and burst I/O architectures, to trade core size against transform time [39]. In addition, commercial FFT IP includes device-specific PPA-oriented optimization techniques. Directly comparing the RTL implementation of the proposed method with such mature IP would mix two types of benefits: the flow-level benefit obtained by replacing full-spectrum reconstruction with selective risk decision and the micro-architecture-level benefit obtained from long-term IP-level engineering optimization. This would introduce unfairness caused by IP-level optimization into the comparison.

For input data, this paper uses Dataset A, Dataset B, and Dataset C. Dataset A is a low-risk steady-state condition. The spectrum has a large margin to the limit curve. Most buckets can be classified directly at the bucket-decision stage. Dataset B is a regulation-transition condition. Some spectral lines are close to the limit boundary. The number of gray-zone buckets increases. More local refinement is needed. This is also the most common condition in an actual closed-loop system. Dataset C is a high-risk stress condition. Near-limit spectral lines, sideband aggregation, or bucket collisions are more obvious. The sequential verification depth increases further. Datasets A to C are used to show the input-dependent latency variation in the proposed design.

Functional verification is performed by cross-checking RTL simulation with the fixed-point software model. Table 3 shows the decision results of the proposed method for the three datasets. Dataset A generates no uncertain bucket. This means that bucket-level fast certification alone completes the decision. Datasets B and C generate 35 and 41 uncertain buckets. They complete 27 and 41 local refinements, respectively. This shows that the proposed implementation can enable or skip the refinement path according to the boundary difficulty of the input spectrum. This is consistent with the selective decision mechanism described in Section 3.3, Section 3.4 and Section 3.5. For the two FFT baselines, the full *N*-point FFT and pointwise limit comparison are always executed. Their latency is almost independent of the input spectrum.

#### 5.2.2. PPA Comparison Analysis

Table 4 summarizes the resource, power, and timing results of the three RTL implementations. The proposed implementation shows its main advantages over the two FFT baselines in BRAM use, dynamic power, and timing margin.

In terms of resources, the proposed implementation uses more LUTs, FFs, and DSPs than the resource-reused FFT. This is because the proposed design adds local decision logic. This includes bucket-level interval decisions, gray-zone bucket management, phase refinement, candidate selection, and sequential verification. This part is the control and arithmetic cost needed to replace full-spectrum recovery with selective decisions. The proposed implementation uses only 17 BRAM tiles. This is much lower than the 352 tiles of the resource-reused FFT and the 704 tiles of the highly parallel FFT. The reductions are about 95.17% and 97.59%, respectively. This shows that the main hardware benefit of the method is lower storage pressure. It avoids large frame buffers, intermediate spectrum storage, and fullband data movement in the full FFT route. In this FPGA prototype, when BRAM usage is migrated to an application-specific integrated circuit (ASIC), it is typically implemented by SRAM macros or register-based memories, depending on storage capacity, port count, bandwidth, and access pattern. Embedded memory studies have reported that embedded memories can occupy more than half of the total die area in a typical SoC [40]. Therefore, the proposed method is expected to provide a certain area advantage in ASIC implementation.

In terms of power, the dynamic power of the proposed implementation is 0.092 W. This is lower than 0.200 W for the resource-reused FFT and 0.542 W for the highly parallel FFT. The reductions are about 54.0% and 83.0%, respectively. The total power difference among the three designs is not large. The main reason is the high static power on the FPGA platform. Dynamic power better shows the energy difference from data-path activity, memory access, and interconnect switching. The proposed implementation uses bucket-level screening and local refinement. It reduces large-scale data movement after full-spectrum computation. It also reduces BRAM access and pointwise comparison activity. This gives a clearer advantage in dynamic power.

In terms of timing, all three designs meet the 100 MHz constraint. The WNS of the proposed implementation is 3.033 ns. This is higher than 1.728 ns for the resource-reused FFT and 0.849 ns for the highly parallel FFT. This result shows that the proposed design has more timing slack at the current RTL level. The proposed method reduces long-distance data movement in the full-spectrum path. This helps timing closure.

To further analyze the relation between performance and power, this paper uses the number of cycles *C* needed to complete one EMI decision. It uses this value to compute the per-decision latency and the per-decision dynamic energy. With clock frequency $f_{clk}$, the per-decision latency is $C/f_{clk}$. The per-decision dynamic energy is $P_{dyn}C/f_{clk}$. The cycle count of the proposed design changes with the decision difficulty of the input. This paper reports the results for Dataset A, Dataset B, and Dataset C separately. It also gives the average.

Table 5 shows the results under the three decision conditions from easy to difficult. The per-decision latencies of the proposed implementation are 4.915 ms, 6.548 ms, and 6.631 ms, respectively. The average latency is 6.031 ms. The number of gray-zone buckets increases from 0 to 35 and 41. The execution cycles also increase. The total still stays below $6.64\times 10^{5}$ cycles.

By comparison, the resource-reused FFT baseline must complete full-spectrum recovery and then do limit comparison. Its per-decision latency reaches 128.451 ms. The highly parallel FFT reduces the latency to 34.079 ms by using a more parallel data path. This value is still much higher than that of the proposed implementation. Based on the average latency, the proposed implementation reduces latency by about 95.30% compared with the resource-reused FFT. It also reduces latency by about 82.30% compared with the highly parallel FFT.

The per-decision dynamic energy also shows the advantage of the proposed design. Its average dynamic energy is 0.555 mJ/decision. In the high gray-zone-bucket case represented by Dataset C, the dynamic energy is still only 0.610 mJ/decision. The per-decision dynamic energies of the resource-reused FFT and the highly parallel FFT are 25.690 mJ/decision and 18.470 mJ/decision. Based on the average value, the proposed implementation reduces energy by about 46.3 times and 33.3 times compared with the two FFT baselines.

It should be noted that the current proposed design is still a preliminary RTL-level implementation. It has not gone through mature IP-level pipelining, memory scheduling, bit-width trimming, or physical-implementation optimization. Future optimization can focus on bucket-level observation pipelining, parallel candidate-bucket refinement, DSP resource reuse, fixed-point bit-width compression, control-path retiming, and layered on-chip storage. These steps can improve the engineering maturity of the proposed design. If the design is moved to an ASIC later, custom SRAM, clock gating, data gating, and dedicated comparison logic can be used. The extra overhead from FPGA-programmable interconnect and static power can also be reduced. The advantages of the proposed method in storage access, dynamic energy, and decision latency are expected to become larger after IP-style or ASIC implementation.

#### 5.2.3. Time-Scale Compatibility with a Slow EMI Risk-Feedback Loop

##### Closed-Loop Interface and Response-Time Interpretation

The average latency of 6.031 ms obtained from the RTL implementation should be interpreted as the digital decision latency of the outer EMI risk-feedback path. For a switching frequency of 100 kHz–1 MHz, 6.031 ms corresponds to approximately 603–6031 switching cycles. This time scale is not intended for cycle-by-cycle power regulation. Instead, it is used to form a stable frequency-domain risk observation over multiple switching events and to update the modulation range of spread-spectrum modulation or excess-spectrum modulation.

This time-scale separation is consistent with existing on-chip closed-loop EMI regulation systems. The GaN power converter reported by Chen et al. has verified a physical closed-loop path consisting of in situ EMI sensing, online spectral assessment, maximum excess-spectrum generation, GESM modulation update, and EMI suppression [4]. In that system, the power-stage output regulation is handled by a fast inner loop, while the EMI regulation loop is designed to be much slower than the output-regulation loop to guarantee multi-loop stability. The same work further states that a sensing/convergence delay of about 10 ms is viable for EMI regulation, and the EMI regulation loop is compressed to the order of 1 kHz by setting an interval of about 1 ms between two $\Delta f_{\mathrm{SW}}$ tuning steps [4]. Therefore, in the context of the on-chip closed-loop EMI regulation architecture already verified by Chen et al., the 6.031 ms average decision latency of this work satisfies the time-scale requirement of frequency-domain risk feedback in a slow EMI outer loop.

The role of this work is to replace and optimize the frequency-domain assessment and risk-variable generation backend in the above closed-loop path. More specifically, this work verifies the low-redundancy replacement feasibility of the digital frequency-domain decision stage in the loop rather than re-implementing a new power-stage closed-loop experiment. In Chen et al.’s system, the maximum excess-spectrum information is obtained through online spectral analysis and then used to adjust the frequency dithering range. In this work, the proposed engine directly generates an internal risk state, a worst-margin estimate, and candidate risk locations from the calibrated sensing-chain output, avoiding full-spectrum reconstruction followed by pointwise limit comparison. In other words, the prior work has verified that on-chip EMI sensing and the GESM actuator can form an effective closed loop, while this work further shows that the frequency-domain risk-decision backend in such a loop can be implemented by a low-redundancy sparse frequency-domain decision engine.

At the system interface, the digital EMI controller can update the modulation range according to the feedback provided by the proposed decision engine. For the *i*-th observation window, the worst-margin estimate is denoted as ${\hat{m}}_{i}$, and a conservative bucket-domain upper bound can also be used. If $r_{i}=\Delta f_{\mathrm{SW},i}/f_{\mathrm{SW},0}$ denotes the modulation-range ratio at the *i*-th update, an outer-loop integral update can be written as

(46) $$r_{i+1}=\mathrm{sat}\left(r_{i}+K_{I}\left({\hat{m}}_{i}-m_{\mathrm{ref}}\right),r_{\min},r_{\max}\right),$$

where $m_{\mathrm{ref}}\leq 0$ is a guard margin, and sat(·) denotes saturation by the allowed modulation range. This equation specifies the interface relationship between the proposed digital decision engine and an RSSM- or GESM-type modulation actuator.

## 6. Conclusions

This paper addresses the computation, storage, and power bottlenecks of the frequency-domain decision backend for resource-constrained EMI risk feedback and proposes a limit-aware selective frequency-domain analysis method. The traditional FFT route first reconstructs the full spectrum and then compares each frequency point with the limit. This paper defines the task as fast extraction of the worst exceedance evidence in the band of interest. The algorithm flow is built around mapped-reference-limit-based internal risk decisions. Specifically, this paper maps the EMI standard limit to a discrete spectral-amplitude threshold and constructs the global worst exceedance margin as the risk-feedback variable for closed-loop regulation. The method combines randomized spectral reordering, flat-window bucket-level observation, bucket-level interval certification, local phase refinement, and sequential worst-case verification, focusing computation on a small number of gray-zone buckets that truly affect the final risk decision.

The projections of measured spectra under no-RSSM and RSSM conditions in the three-dimensional degradation space also show the usable range of the method. The target GaN switching power supply EMI spectra lie inside the usable region, indicating the engineering applicability of the proposed method.

RTL-level PPA evaluation shows clear improvements over the two FFT baselines. The average per-decision latency is reduced by about 95.30% and 82.30%, BRAM usage is reduced by about 95.17% and 97.59%, dynamic power is reduced by about 54.0% and 83.0%, and the average per-decision dynamic energy is reduced by about 46.3 times and 33.3 times. These results show that the proposed method reduces storage access, data movement, and unnecessary computation caused by full-spectrum reconstruction and provides a low-redundancy, low-energy, and timing-friendly implementation path for the frequency-domain decision backend used in internal EMI risk feedback in resource-constrained systems.

Future work can continue in three directions: robustness enhancement, sensing-chain calibration, and system-level closed-loop validation. First, for extremely complex EMI spectra that exceed the applicability boundary discussed above, a *K*-adaptive budget-allocation mechanism can be introduced, and the effects of different non-dominant spectral components on the uncertain-bucket ratio, local refinement depth, and risk-decision consistency can be further studied to broaden the applicability boundary of the algorithm. Second, a more accurate transfer relationship, error boundary, and correlation model between the output of an on-chip or in situ EMI sensing chain and standard EMI-receiver measurements need to be established on an experimental platform. This would clarify the engineering relationship between the internal risk variable used in this work and product-level conducted EMI emissions. Third, a continuous-input streaming verification system can be built on an FPGA or ASIC prototype. This system can connect the sampling buffer, bucket-level observation, limit mapping, gray-zone refinement, risk-variable output, and control interface to verify the timing behavior and stability of the method in an online risk-feedback path. Finally, complete closed-loop modulation experiments can be carried out on a real GaN power converter platform. The EMI spectra before and after control, the temporal trajectory of the risk variable, the modulation-parameter response, and the trade-offs among efficiency and output ripple should be recorded. Through these steps, the limit-aware frequency-domain decision framework proposed in this paper can move from offline internal risk decision toward an integrated risk-feedback unit for on-chip EMI management.

## Figures and Tables

**Table 1:** EN 55032 Class B conducted-emission quasi-peak reference values used for internal EMI risk-decision experiments.

Frequency Range	Limits (dBμV) (Quasi-Peak)
MHz	
0.15 to 0.50	66 to 56
0.50 to 5	56
5 to 30	60

**NOTE 1** The lower limit shall apply at the transition frequencies. **NOTE 2** The limit decreases linearly with the logarithm of the frequency in the range 0.15 MHz to 0.50 MHz.

**Table 2:** Summary statistics of risk-positive-triggering complexity for different ordering strategies under worst-case sequential verification.

Method	Mean	Median	90th pct	P(T≤5)
LB	18.45	20.00	36.00	30.1%
MID	23.98	25.00	54.00	43.0%
UB	24.40	26.00	55.00	43.1%
|Y|	18.53	4.00	54.00	59.3%
Random	21.05	19.00	41.00	16.6%

Red values indicate the best result for each metric; lower values are better for Mean, Median, and 90th pct, whereas a higher value is better for P(T≤5).

**Table 3:** Functional simulation results of the proposed implementation.

Dataset	Decision	Gray-Zone Buckets	Refined Buckets	Cycles
A	risk-negative	0	0	491,538
B	risk-positive	35	27	654,845
C	risk-negative	41	41	663,063

**Table 4:** Resource, power, and timing results of the three implementations.

Metric	Resource-Reused FFT	Highly Parallel FFT	Proposed Implementation
BRAM	352	704	17
FF	282	585	1500
LUT	1511	4211	2670
DSP	8	8	32
$P_{\mathrm{dyn}}$/W	0.200	0.542	0.092
WNS/ns	1.728	0.849	3.033

Red values indicate the proposed implementation. They are highlighted to distinguish the proposed design from the two FFT baseline implementations.

**Table 5:** Latency and dynamic energy per decision.

Implementation	Cycles	Latency/ms	$E_{\mathrm{dyn}}$/mJ
Proposed-A	491,538	4.915	0.452
Proposed-B	654,845	6.548	0.602
Proposed-C	663,063	6.631	0.610
Proposed-Mean	603,149	6.031	0.555
Resource-reused FFT	12,845,058	128.451	25.690
Highly parallel FFT	3,407,892	34.079	18.470

Red values indicate the mean result of the three proposed implementation cases.

## Data Availability

The original contributions presented in this study are included in the article. Further inquiries can be directed to the corresponding author.
